# A high-fat eucaloric diet induces reprometabolic syndrome of obesity in normal weight women

**DOI:** 10.1093/pnasnexus/pgad440

**Published:** 2023-12-18

**Authors:** Nanette Santoro, Katherine Kuhn, Shannon Pretzel, Irene E Schauer, Angela Fought, Angelo D’Alessandro, Daniel Stephenson, Andrew P Bradford

**Affiliations:** Department of Obstetrics and Gynecology, University of Colorado School of Medicine, Aurora, CO 80045, USA; Department of Obstetrics and Gynecology, University of Colorado School of Medicine, Aurora, CO 80045, USA; Department of Obstetrics and Gynecology, University of Colorado School of Medicine, Aurora, CO 80045, USA; Department of Medicine, Rocky Mountain Regional VA Medical Center, Aurora, CO 80045, USA; Division of Endocrinology, Metabolism, and Diabetes, University of Colorado School of Medicine, Aurora, CO 80045, USA; Department of Biostatistics and Informatics, Colorado School of Public Health, Aurora, CO 80045, USA; Department of Biochemistry and Molecular Genetics, University of Colorado School of Medicine, Aurora, CO 80045, USA; Department of Biochemistry and Molecular Genetics, University of Colorado School of Medicine, Aurora, CO 80045, USA; Department of Obstetrics and Gynecology, University of Colorado School of Medicine, Aurora, CO 80045, USA

**Keywords:** high-fat diet, eucaloric, LH, FSH, gonadotropin suppression

## Abstract

We examined the effects of 1 month of a eucaloric, high-fat (48% of calories) diet (HFD) on gonadotropin secretion in normal-weight women to interrogate the role of free fatty acids and insulin in mediating the relative hypogonadotropic hypogonadism of obesity. Eighteen eumenorrheic women (body mass index [BMI] 18–25 kg/m^2^) were studied in the early follicular phase of the menstrual cycle before and after exposure to an HFD with frequent blood sampling for luteinizing hormone (LH) and follicle-stimulating hormone (FSH), followed by an assessment of pituitary sensitivity to gonadotropin-releasing hormone (GnRH). Mass spectrometry-based plasma metabolomic analysis was also performed. Paired testing and time-series analysis were performed as appropriate. Mean endogenous LH (unstimulated) was significantly decreased after the HFD (4.3 ± 1.0 vs. 3.8 ± 1.0, *P* < 0.01); mean unstimulated FSH was not changed. Both LH (10.1 ± 1.0 vs. 7.2 ± 1.0, *P* < 0.01) and FSH (9.5 ± 1.0 vs. 8.8 ± 1.0, *P* < 0.01) responses to 75 ng/kg of GnRH were reduced after the HFD. Mean LH pulse amplitude and LH interpulse interval were unaffected by the dietary exposure. Eucaloric HFD exposure did not cause weight change. Plasma metabolomics confirmed adherence with elevation of fasting free fatty acids (especially long-chain mono-, poly-, and highly unsaturated fatty acids) by the last day of the HFD. One-month exposure to an HFD successfully induced key reproductive and metabolic features of reprometabolic syndrome in normal-weight women. These data suggest that dietary factors may underlie the gonadotrope compromise seen in obesity-related subfertility and therapeutic dietary interventions, independent of weight loss, may be possible.

**Significance Statement**

This work confirms that gonadotropin secretion can be dysregulated in normal weight, normally cycling women by a 1-month exposure to a high-fat diet. The dietary exposure mimics the endocrine and metabolic milieu of obesity and implies that dietary factors may be responsible for the reproductive impairments observed in women with obesity.

## Introduction

Obesity exerts several detrimental effects on reproduction. Increased female body mass index (BMI) is linked to a longer time to conception (1, 2), lower live birth rates or higher cancelation risk after assisted reproductive procedures in most (3–6), but not all (7) studies, and a greater risk for pregnancy loss (8). Mechanisms responsible for the reduced fecundity and poorer reproductive performance of women with obesity are not fully known, but decreased gonadotropin and sex steroid production (9–12), along with reduced inhibin B (13) have all been reported.

It is important to understand the mechanisms that bring about obesity-related infertility because obvious solutions such as weight loss interventions are not effective in increasing live birth rates in women with obesity who do not have polycystic ovary syndrome (PCOS) (14, 15). Despite some improvements in metabolic parameters postweight loss and inconsistent maternal benefit of weight loss (16, 17), fertility was not improved in either of these two major, well-powered, and effective randomized clinical trials that each resulted in >5% weight loss.

We have previously reported that women with obesity demonstrate reduced luteinizing hormone (LH) pulse amplitude and decreased luteal progesterone metabolite excretion (11) which is partially reversed with surgical weight loss (18). We have called the condition of non-PCOS “simple” obesity in association with relative, uncompensated hypogonadotropic hypogonadism “reprometabolic syndrome.” To attempt to isolate factors that could contribute to the aberrant hormonal milieu in women with high BMI, we examined gonadotropin dynamics in normal-weight women administered either insulin, lipid infusion, or both and compared these to a saline infusion (19). The combination of short-term (6-h) infusion of both insulin and lipid led to suppression of gonadotropins in a small sample of men and women. In follow-up experiments, we performed frequent blood sampling in a sample of 15 normal-weight women in the early follicular phase of the menstrual cycle with and without an infusion of insulin and lipid, and demonstrated a partial induction of reprometabolic syndrome, with significantly reduced follicle-stimulating hormone (FSH) secretion and LH response to gonadotropin-releasing hormone (GnRH) (20). These effects were not accompanied by any clinically meaningful change in inflammatory markers (21) or other pituitary hormones (22).

**Fig. 1.**
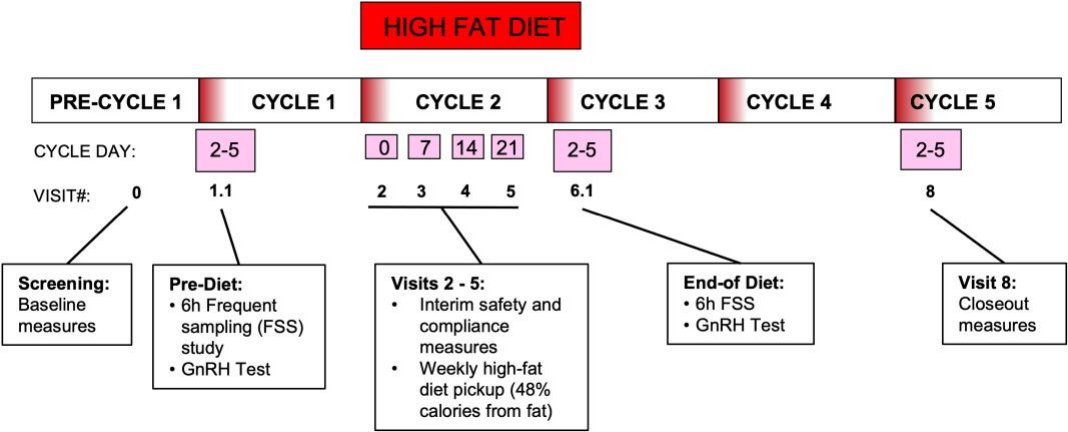
Schematic of the protocol. Participants underwent a screening visit for eligibility and completed a 3-day food record to assess food preferences and estimate fat consumption. In the early follicular phase of cycle 1, a 6-h frequent sampling study was performed including a 75 ng/kg GnRH bolus in the final 2 h of sampling. Throughout cycle 2, the high-fat diet was administered with weekly blood sampling for compliance and safety checks. The diet was continued until the early follicular frequent sampling study was completed in the early follicular phase of cycle 3. Cycle 4 was a recovery cycle and at the end of cycle 5 participants had a final closeout visit with a repeat of safety laboratory tests.

To test whether a combination of exposure to high-fat and excess insulin could reproduce features of reprometabolic syndrome in normal-weight women under “real-life” conditions, we exposed a group of 18 normally cycling women with a BMI between 18 and 25 kg/m^2^ to ∼1 month of a eucaloric diet containing 48% calories from fat. We studied gonadotropin and sex steroid patterns before, during, and at the end of the dietary exposure and conducted a detailed metabolomic analysis to assess the nonreproductive effects of the dietary exposure. A schematic of the study design is shown in Fig. 1.

## Results

### Characteristics of the study sample

The final sample of women who completed all assessments was 18. Their baseline characteristics are presented in Table 1. Mean anti-Mullerian hormone (AMH) was normal (3.18 ± 4.3) but substantial variation was observed, including three participants with values <1.0. Other parameters are consistent with a young, healthy population, and inclusion criteria.

**Table 1.** Baseline characteristics of the study sample.

Parameter	Participants
Enrollment	18
Age (year)	29.7 (±5.9)
BMI (kg/m^2^)	21.6 (±2.0)
Weight (kg)	60.1 (±9.7)
Height (cm)	166.7 (±9.1)
Cycle length (days)	28.3 (±2.2)
TSH (mIU/mL)	1.84 (±1.1)
HbA1c (%)	5.05 (±0.2)
Prolactin (ng/mL)	12.6 (±6.4)
AMH	3.3 (±3.9)

Data shown are mean ± SD.

### LH response to the high-fat diet

**Fig. 2.**
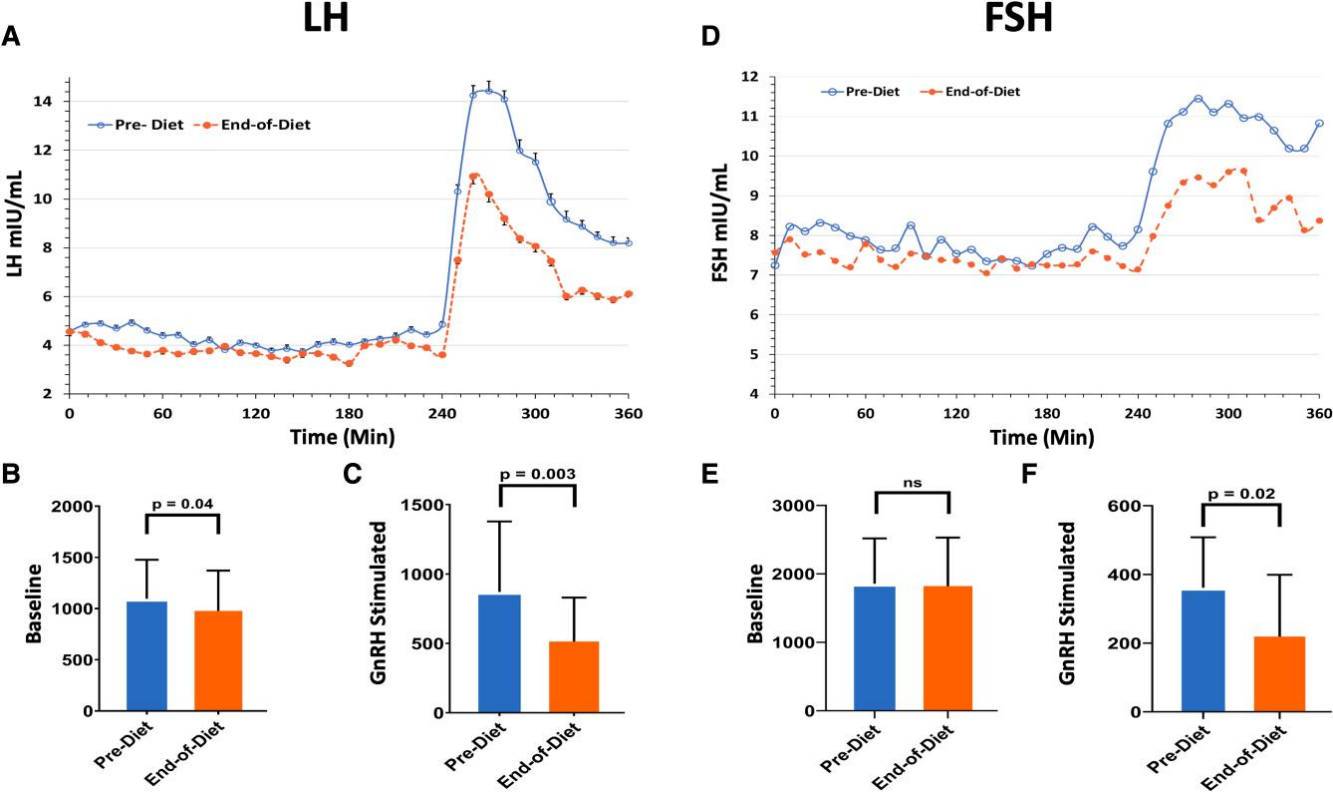
Early follicular phase gonadotropin response to the high-fat diet. A) LH levels were measured during the frequent blood sampling session before and at the end of the high-fat diet administration. Data are mean ± SEM. The open circles are before the diet was administered and the closed circles are the end-of-diet study. B) The area under the curve (arbitrary units) for baseline endogenous LH sampling and C) the GnRH-stimulated LH response. D) FSH levels during the pre-diet and end-of-diet frequent blood sampling sessions. The open circles represent the prediet visit, and the closed circles are the end-of-diet visit. Data are shown as mean ± SEM. E) Area under the curve (arbitrary units) for baseline endogenous FSH and F) GnRH-stimulated FSH response. *P*-values were determined by paired t test.

Mean LH was significantly decreased after the high-fat diet (4.3 ± 1.0 vs. 3.8 ± 1.0, *P* < 0.01) as was LH response to exogenous GnRH (10.1 ± 1.0 vs. 7.2 ± 1.0, *P* < 0.01; Table 2 and Fig. 2A–C). The mean LH pulse amplitude was 2.4 ± 1.1 (SD) IU/L at baseline and did not change after exposure to the high-fat diet (2.5 ± 1.3, *P* = 0.94). There was no difference in LH interpulse interval before the high-fat diet (83 ± 11 min [SEM]) compared with afterward (85 ± 11 min).

**Table 2.** Comparison of reproductive parameters prediet and end-of-diet.

Parameter	Pre-diet	End-of-diet	*P-*value
Height (m)	1.7 (±0.1)	1.7 (±0.1)	0.42
Weight (kg)	60.1 (±9.7)	59.8 (±9.3)	0.46
BMI (kg/m^2^)	21.6 (±2.0)	21.4 (±1.8)	0.33
LH pulse amplitude (IU/L)	2.4 (±1.1)	2.5 (±1.3)	0.94
LH pulse frequency (min)	83 (±11)	85 (±1.1)	0.85
Mean LH (IU/L)	4.3 (±1.0)	3.8 (±1.0)	<0.01
LH response to GnRH^a^	10.1 (±1.0)	7.2 (±1.0)	<0.01
Mean FSH (IU/L)	7.8 (±1.0)	7.4 (±1.0)	0.08
FSH response to GnRH^a^	9.5 (±1.0)	8.8 (±1.0)	<0.01

Data shown are mean ± SD. ^a^Data are shown as area under the curve and arbitrary units.

### FSH response to the high-fat diet

Mean FSH did not differ significantly at baseline after exposure to the high-fat diet (7.8 ± 1.0 vs. 7.4 ± 1.0, *P* = 0.08). FSH response to exogenous GnRH was significantly blunted (9.5 ± 1.0 vs. 8.8 ± 1.0, *P* < 0.01) at the end of the high-fat diet exposure (Table 2 and Fig. 2D–F).

### Change in participant status over time

Weight did not change in participants over the course of the investigation nor did menstrual cycle length. Estradiol (34.8 ± 14.7 pg/mL and 32.7 ± 19.7 pg/mL) and sex hormone binding globulin (SHBG) (53.9 ± 25.6 nmol/L and 46.8 ± 19.6 nmol/L) did not differ between the pre and end-of-diet frequent sampling studies. Red blood cell fatty acid profiles (Fig. S1A) indicated an overall increase in measured fatty acids compared prediet to end-of-diet. Significant increases were observed for palmitoleic acid, docosahexaenoic acid (DHA), eicosapentaenoic acid (EPA), and dihomo-γ-linolenic acid (DGLA). Lipidomic profiles (Fig. S2) also indicated significant trends for phosphatidylcholine and sphingomyelin. Table 3 indicates changes in study lipid parameters before and after the month of high-fat feeding. No changes in urinary ketones were observed in response to the high-fat diet and no participant exhibited any signs of ketosis.

**Table 3.** Comparison of fasting lipid parameters prediet and end-of-diet.

Parameter	Before diet	End-of-diet
Cholesterol (mg/dL)	164.8 (±31.5)	153.35 (±29.7)
Low-density lipoprotein (LDL) (mg/dL)	90.8 (±27.7)	90.24 (±31.5)
High-density lipoprotein (HDL) (mg/dL)	58.2 (±10.4)	50.71 (±31.5)
Triglycerides (mg/dL)	82.4 (±40.0)	63.12 (±31.5)

Data shown are mean ± SD.

### Plasma metabolomics

**Fig. 3.**
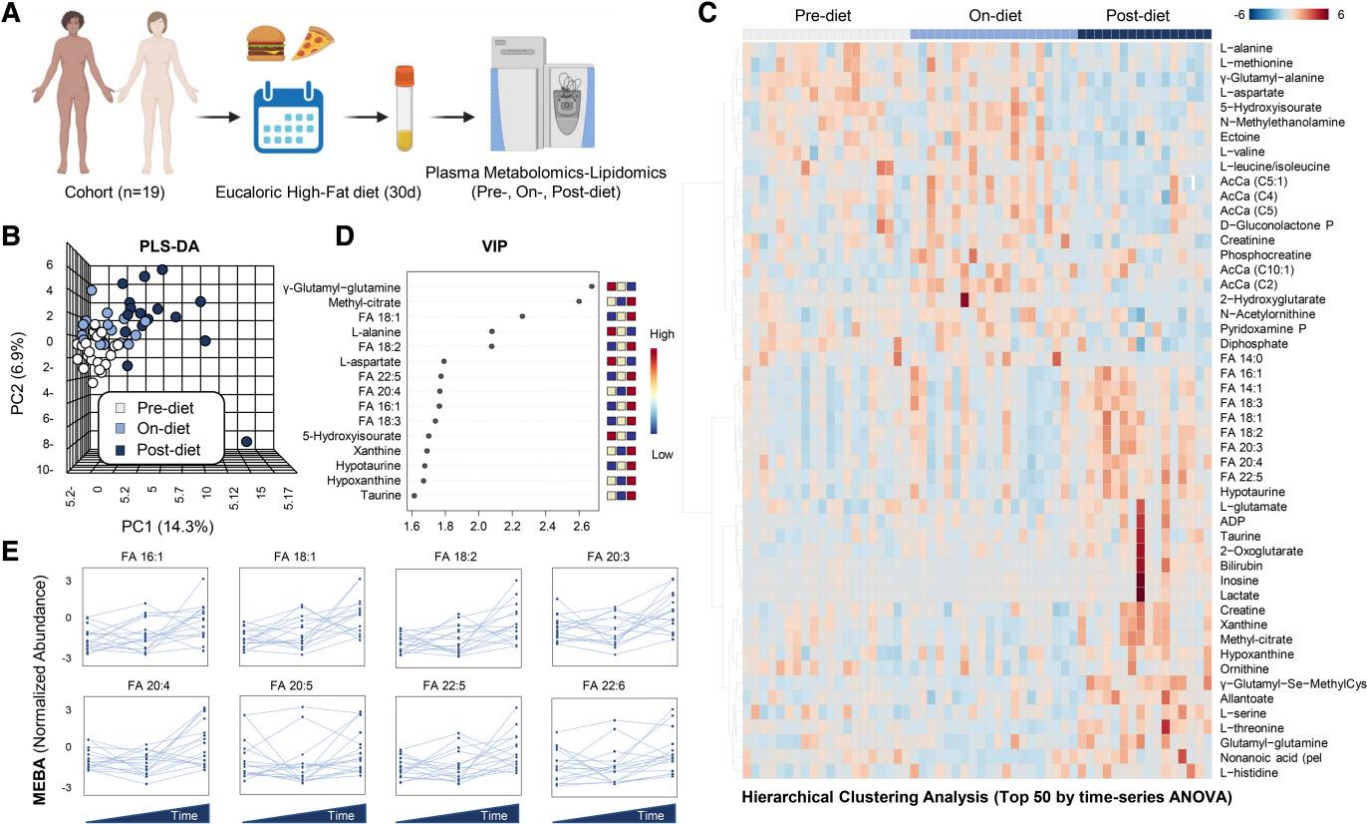
To assess compliance with the diet we performed lipidomic and metabolic analysis on plasma collected before, during, and at the end of the high-fat diet exposure. A) Describes the protocol; samples were analyzed before, during, and at the end of the high-fat diet, which was given for ∼30 days (beginning of one menstrual cycle through the subsequent menstrual cycle's frequent sampling study in the early follicular phase). B) PLS-DA indicates clear-cut differences in principal components at the three time points. C) HCA of the top 50 significant features by repeated measures ANOVA indicates diet-related depletion of amino acids (methionine, valine, alanine, aspartate, and leucine/isoleucine) and increases in carnitine and multiple fatty acids, carboxylic acids, bilirubin, taurine, and hypotaurine. D) Variable importance in projection analysis highlights the features with the highest loading weights from the PLS-DA elaboration. The top 20 metabolites informing the clustering include free fatty acids, purine deamination and oxidation products, and taurine metabolites. E) Line plots (each independent line illustrates the trajectories for each different subject) for free fatty acids indicate increases in association with exposure to the high-fat diet.

Metabolomics analyses were performed on plasma from all participants at baseline (prediet), while on diet and at the end-of-diet (Fig. 3A). Multivariate analyses of metabolomics data show a clear progression in the plasma metabolome over time, as gleaned by partial least squares-discriminant analysis (PLS-DA)—which discriminated samples across principal component 1 (explaining 14.3% of the total variance—Fig. 3B). Hierarchical clustering analysis (HCA) of metabolomics data revealed three main trends: (i) a progressive depletion of several amino acids (including methionine, valine, alanine, aspartate, leucine/isoleucine); (ii) a transient increase of acyl-carnitines while on diet (AcCA C4, 5, 5:1, 10:1); and (iii) increases during and at the end-of-diet of multiple fatty acids (saturated: 14:0, 16:0; monounsaturated: 16:1, 18:1, poly and highly unsaturated: 18:2, 18:3, 20:3, 20:4, 22:5, and 22:6), carboxylic acids (2-oxoglutarate, methyl-citrate, 2-hydroxyglutarate, and lactate), bilirubin, taurine, and hypotaurine (Fig. 3C). Variable importance in projection (VIP—Fig. 3D) identified the top 20 metabolites informing such clustering, which included a series of free fatty acids (FA) (16:1, 18:1, 18:2, 18:3, 20:4, and 22:5), purine deamination, and oxidation products (hypoxanthine, xanthine, and 5-hydroxyisourate), and taurine metabolites (taurine, hypotaurine). Line plots for free fatty acids are consistent with adherence to the eucaloric high-fat diet (Fig. 3E).

**Fig. 4.**
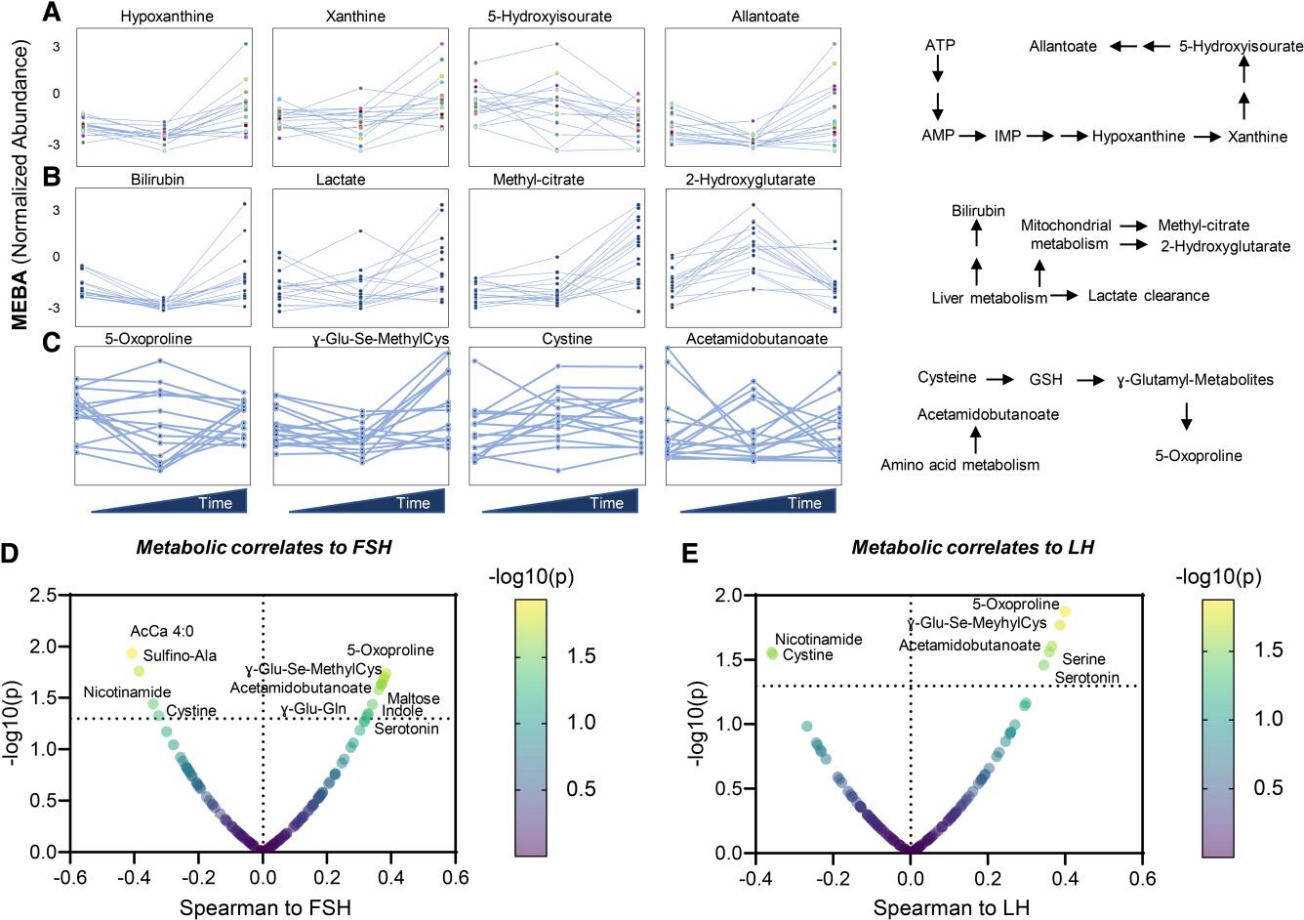
Metabolic pathways affected by the high-fat diet. Line plots (each independent line illustrates the trajectories for each different subject) for purine deamination and oxidation A), carboxylic acid metabolites B), and glutathione-related metabolites C), in association with the high-fat diet. A summary overview of the main metabolic pathways affected by the treatment is shown to the right of the plots. D) Metabolic pathways are significantly and strongly associated with FSH D) and LH E), as gleaned by Spearman correlation analyses.

Significant trends through the duration of the trial were observed for purine deamination and oxidation (Fig. 4A), carboxylic acid metabolites (Fig. 4B), and metabolites involved in glutathione homeostasis, the gamma-glutamyl-cycle, and amino acid catabolism (Fig. 4C). Notably, despite heterogeneous trends across the sample of women enrolled in the study, the latter group of metabolites demonstrated the strongest, significant positive correlation to LH and FSH levels through the trial (Fig. 4D and E).

The correlation of metabolomics data to nutritional data on dietary composition throughout the trial confirms a strong correlation between dietary intake and small molecule metabolite levels in plasma (Fig. S1A). Debiased sparse correlation network analysis of combined metabolomics and dietary composition data confirmed a strong correlation between circulating fatty acids and amino acids (Fig. S1B).

## Discussion

Herein we demonstrate that a 1-month exposure to a high-fat diet, containing ∼48% calories from fat, is capable of inducing gonadotropin suppression in normal-weight women and reproducing the aberrant gonadotropin dynamics observed in women with obesity. While LH pulsatility was not suppressed, mean LH and the response of both LH and FSH to GnRH were significantly reduced after the high-fat diet. These characteristics of reprometabolic syndrome, particularly the reduced pituitary response to GnRH, are abiding features of obesity-related hypothalamic-pituitary axis dysfunction (11, 23). We further demonstrate that the high-fat exposure resulted in multiple significant changes in the metabolome, indicative of increased metabolic stress imposed by the diet. The fact that we were able to induce these changes with a “real-life” limited duration exposure to high-fat feeding, without weight change, strongly implies that dietary intake and circulating lipids play a role in the relative hypogonadotropic hypogonadism of obesity.

Lipid exposure has been examined as a cause of gonadotrope dysfunction in some animal models. Li et al. (24), implicated oleate as a specific inhibitor of LH beta subunit expression in mouse gonadotropes. Inhibition of gonadotropin gene expression appeared related to the induction of endoplasmic reticulum (ER) stress in this system. In our prior, short-term infusion studies noted above, we did not observe any increase in circulating markers of ER stress (21); however, pituitary ER stress may not result in detectable systemic changes in such markers. Alterations of circulating levels of acyl-carnitines and free fatty acids have been recently reported in the context of hypothalamic-pituitary-gonadal axis inhibition in longitudinal samples from transgender males (assigned females at birth and undergoing gender reassignment therapy). Here, similar changes in circulating acyl-carnitines and free fatty acids were observed. Most high-fat diet experiments in animal models involve hypercaloric feeding, which results in weight gain, increased inflammation, and elevated adipocytokines. However, we observed neuroendocrine changes in the absence of weight gain and without increases in multiple circulating inflammatory markers. Metabolomics data showed an effect of the high-fat eucaloric diet on circulating levels of free fatty acids, increasing over time, high-energy purine breakdown, and deamination products. These metabolites are usually associated with increased deaminase activity as a function of increased oxidant stress (25), or altered mitochondrial metabolism as a function of hypoxia (26). In this context, it is worth noting that circulating levels of carboxylic acids lactate (27, 28), methyl-citrate (29), 2-hydroxyglutarate (30), and 2-oxoglutarate (31), are all consistent with signatures of mitochondrial dysfunction. Similarly, bilirubin accumulation (usually inversely correlated to the onset of nonalcoholic fatty liver disease) (32) and dysregulation of taurine metabolism (33) are suggestive of a moderate impact of the diet on liver metabolism. The pituitary gland lies outside of the blood–brain barrier and is thus exposed to the peripheral circulation. It therefore seems likely that lipid exposure itself is exerting a selective effect on gonadotropes, but the precise mechanism by which this effect is occurring is currently not known.

In prior work, we have noted relatively large decrements, of ∼50%, in basal LH and FSH secretion in women with obesity and reprometabolic syndrome, and reductions of GnRH-stimulated LH and FSH of ∼30%. In this study, we found a similar magnitude of change of GnRH-stimulated LH (29%) but a lesser deficit in GnRH-stimulated FSH (8%) and smaller differences in mean LH levels from prediet to end-of-diet for both hormones (12 and 5%, respectively). Thus, the model does not perfectly reproduce reprometabolic syndrome of obesity, indicating that additional features of chronic obesity may lead to uncompensated changes that impact gonadotropin secretion. The fact that our participants were all studied in the fasting state when their lipid and lipoprotein profiles did not differ between baseline and end-of-diet, may imply that there is a relatively acute effect of nutritional excess that is related to reprometabolic syndrome or that, in the case of obesity and chronic overfeeding, gonadotropin secretion is somehow reset at a lower level. To this end, studies of normal weight women in both the fed and fasting state might be revealing.

We have previously demonstrated that short-term exposure to a 6-h infusion of a lipid emulsion plus insulin caused a partial recapitulation of reprometabolic syndrome in a sample of normal-weight women, studied in the early follicular phase of their menstrual cycles (20), as well as a mixed sample of men and women (19). The effect we noted in these earlier studies was rapid, and evidence of gonadotropin suppression was observed within a few hours of exposure. Investigation of adipocytokines and other inflammatory markers (21) in this short-term model, including interleukin (IL)-6 and IL-12, monocyte chemotactic protein-1 (MCP-1), tumor necrosis factor (TNF)α and β and C-reactive protein (CRP) indicated no differences between saline infusion and lipid plus insulin infusion; only macrophage inflammatory protein-1β (MIP-1β) demonstrated a statistically significant increase (*P* = 0.03) after lipid plus insulin infusion. Circulating markers of endoplasmic reticulum stress, such as CCAAT-enhancer-binding protein (C/EBP) homologous protein transcription factor (CHOP) and glucose-regulated protein (GRP 78), and the metabolic regulator fibroblast growth factor (FGF) 21 was similarly unchanged by lipid plus insulin infusion. In the same women, we examined a number of nonreproductive pituitary hormones in response to acute hyperlipidemia and hyperinsulinemia and observed no significant differences in levels of thyroid-stimulating hormone (TSH), prolactin, growth hormone, insulin-like growth factor (IGF)-1, or creatinine, although a small but statistically significant increase in leptin was noted (22). Taken together, the data favor a direct and selective effect of high-fat exposure on gonadotropes.

This study builds upon prior work by demonstrating that chronic exposure to elevated triglycerides and free fatty acids through the dietary intake is capable of faithfully reproducing reprometabolic syndrome, as both LH and FSH response to GnRH was impaired in this study, along with mean LH. While the dynamics of fatty acid exposure are admittedly different with a high-fat diet compared with direct lipid infusion, both result in overall increased exposure to triglycerides and free fatty acids. The data further point toward a diet-induced deficit selective for gonadotropins in reprometabolic syndrome, since other studies by our group have shown that only LH and FSH are impacted by free fatty acid infusion with insulin and that both exposure to free fatty acids and insulin together are required for this effect (19). Thus, while other defects at the level of the ovary and possibly endometrium have been described as potential contributors to obesity-related infertility, there appears to be a significant and substantial contribution to chronic gonadotropin insufficiency. Our data indicate a defect in pituitary response to GnRH; however, since GnRH cannot be directly measured, we are unable to ascertain whether obesity is accompanied by an additional deficit in GnRH secretion.

Our goal was to have participants adhere to a high-fat diet that supplied no additional calories and therefore caused them to remain weight neutral. Compliance with the high-fat diet was high as evidenced by the changes observed in a variety of metabolic markers, which was a secondary, hypothesis-generating goal of this study. Few participants reported difficulty maintaining adherence to the diet. All participants returned unused food in order to estimate their caloric intake and little unused food was returned. Most participants reported that the diet was very satiating. One participant with relatively high levels of physical activity reported hunger on the high-fat diet and was encouraged to consume high-fat snacks, such as peanut butter until more food could be supplied. Somewhat surprisingly, we did not observe changes in routine serum lipids and lipoproteins before and at the end of the high-fat feeding, although expected changes were observed in the lipidomic analysis. However, participants always presented in the fasted state for frequent sampling studies and blood draws and were relatively young and healthy. It may take a more pronounced and prolonged dietary challenge to create fasting dyslipidemia in such a sample.

Strengths of this study include the paired design, the detailed and well-timed sampling protocols, and the high level of adherence to the diet, as confirmed by mass spectrometry-based approaches. The level of rigor of the protocol was high and participants were contacted frequently by the study team to assure that any barriers to compliance were addressed. Additional assessments, not included in this report, are pending to determine the effect of the diet on day-to-day hormone production and insulin sensitivity. Weaknesses of the study include the relatively small sample size and the ∼1-month duration of the diet. Because the diet duration was relatively short, we cannot rule out compensatory changes in pituitary or ovarian function that might occur over longer periods of time. Our primary outcome was LH pulse amplitude, and this was not met. However, over the course of the completion of these studies, the initial assays used to demonstrate LH pulse characteristics, DELFIA (Wallac, Turku, and Finland), were no longer available for use in the United States and we adapted the study to the Siemens Advia Centaur. Use of this assay did not provide the same resolving power for LH pulsatility at the relatively low levels observed in this sample and, therefore, we relied upon mean LH and FSH levels as being more representative of the condition of the participants. Mean endogenous (non-GnRH-stimulated) LH was definitively decreased by the high-fat diet but the effect on mean endogenous FSH was of marginal significance (*P* = 0.08). This may reflect the overall small sample size or the relatively large variation in ovarian reserve (as measured by AMH) in the study sample, which can lead to erratic changes in FSH independent of the dietary intervention. LH pulse amplitude has been previously observed by our group to be markedly dampened in women with obesity compared to women of normal BMI (11, 18). We cannot be certain that the observed lack of effect on LH pulse amplitude reflects differences in the mechanism of gonadotropin suppression in normal-weight women exposed to a high-fat diet as compared with obesity; it is also possible that it is simply due to the methodological limitations of the LH measurements.

In summary, we have demonstrated that the reproductive phenotype of obesity, reprometabolic syndrome, can be recreated in normal-weight women with the consumption of a eucaloric, high-fat diet for as little as 1 month duration. Reduced pituitary production of LH and reduced response of both LH and FSH to a physiologic, weight-based dose of GnRH were reproduced, and imply that dietary factors that are circulating in the bloodstream may be the underlying cause of the condition. Further studies are needed to determine whether dietary adjustments, independent of weight loss, can reverse reprometabolic syndrome in women with obesity, and thereby improve fertility.

## Materials and methods

### Participants

This study was a clinical trial (NCT02653092) and was approved by the Colorado Multiple Institutional Review Board (COMIRB). All participants provided informed consent. Women aged 18–38 with menstrual cycles 25–35 days in length and no use of reproductive hormones within 3 months of enrollment were eligible for the study. Additional eligibility criteria included: (i) no history of chronic disease affecting hormone production, metabolism, or clearance and no use of medications known to interact with reproductive hormones or insulin metabolism; (ii) normal screening prolactin and TSH; (iii) normal hemoglobin A1c; (iv) hemoglobin > 11 g/dL at screening; (v) use of reliable nonhormonal contraception throughout the study period. Because the goal was to investigate the effects of a high-fat diet, women with a baseline dietary assessment indicative of >40% calories from fat were excluded, as were women who were unable to comply with the protocol, which involved eating only foods provided by the Colorado Clinical and Translational Sciences Institute's (CCTSI) Nutrition Services. Because of the restrictions on their ability to consume animal or dairy fats, vegans, and lactose-intolerant individuals were excluded.

### Sequence of events

Cycle 1 was a baseline cycle; cycle 2 entailed consumption of the high-fat diet which was continued until frequent blood sampling was completed in the early follicular phase of cycle 3. Cycle 3 was the immediate posthigh-fat diet cycle and cycle 4 was a recovery cycle (Fig. 1). Safety laboratory tests were repeated at the end of cycle 5.

Participants underwent baseline and end-of-diet assessments of gonadotropin dynamics. A 6-h frequent blood sampling study was performed to determine endogenous LH and FSH secretion (time 0–240 min) and concluded with a physiologic bolus of 75 ng/kg GnRH to assess pituitary responsiveness (time 240–360 min). Frequent sampling studies for gonadotropin determination were all performed in the early follicular phase of the menstrual cycle (cycle days 2–5). Participants were also seen weekly during the high-fat diet cycle and had a blood draw to assess compliance.

### High-fat diet protocol

Participants completed a food preference screening survey and a 3-day diet diary, which estimated their daily caloric intake and approximate proportion of daily calories derived from fat. Participants were provided with a customized eucaloric high-fat diet comprised of ∼48% calories from fat, 33% calories from carbohydrates, and 19% calories from protein. Food was portioned into individual meals with snacks and boxed into 3–4 days’ worth at a time. Participants picked up food directly from the Clinical and Translational Research Center (CTRC); when this was not possible, study staff delivered food to the participants. Urinary ketones were measured at each frequent blood sampling session (Siemens Healthineers, Malvern, PA, USA).

### Frequent blood sampling protocol

Participants were instructed to report to the Colorado CCTSI's CTRC within days 2–5 after the onset of menses for an 8 am to 2 pm blood sampling session. An intravenous line was placed and 3 mL blood samples were drawn every 10 min for 6 h. The first 4 h were sampled with no other medications; at 240 min, a 75 ng/kg bolus GnRH (gonadorelin, Ferring, Parsippany, NJ, USA; IND 11878) was administered intravenously over ∼1 min, and sampling was continued for another 2 h. At 6 h the intravenous line was removed, and sampling was concluded.

### Additional measures

During the administration of the high-fat diet, blood samples were drawn weekly for metabolomic analysis to determine compliance with the protocol. Body composition was assessed by DXA (Horizion W 300734N; Hologic Inc., Malborough, MA, USA) at the beginning (visit 1.1) and completion of the protocol (visit 6.1) at the CTRC. Activity and sleep were estimated using a Fitbit device which was given to all participants. Body composition and activity data are the topic of a separate report.

### Hormone measurements

Blood samples from the q10′ sampling (3 mL) were stored overnight at 4°C and centrifuged the following morning to separate serum. If assays were not run immediately after the overnight separation, serum was stored at −80^°^C until thawed for assay. A 20-µL aliquot from each time point was withdrawn from the serum samples before freezing to create a pool for the entire study.

LH, FSH, estradiol, and SHBG were measured using a competitive chemiluminescent immunoassay (Advia Centaur XP; Siemens Healthcare Diagnostics). Inter- and intra-assay coefficient of variations (CVs) were 4.8 and 3.4% for LH, respectively, and 6.6 and 5.0% for FSH, respectively. AMH was measured using the Ansh picoAMH enzyme linked immunosorbent assay (ELISA) (Ansh Labs, Webster, TX, USA), which has a sensitivity of 0.023 ng/mL and inter- and intra-assay CVs of 3.7–8.1 and 2.5–5.5%, respectively.

### Metabolomics

Plasma for metabolomic measurement was obtained after an overnight fast and 20 μL was extracted in 980 μL of methanol:acetonitrile:water (5:3:2, v/v/v). After vortexing at 4°C for 30 min, extracts were separated from the protein pellet by centrifugation for 10 min at 18,000*×g* at 4°C and stored at −80°C until analysis.

Metabolomic analyses were performed using a Vanquish ultra-high-performance liquid chromatography (UHPLC) coupled online to a Q Exactive mass spectrometer (Thermo Fisher, Bremen, Germany). Samples were analyzed using a 5-min gradient as described (34–36). Solvents were supplemented with 0.1% formic acid for positive mode runs and 1 mM ammonium acetate for negative mode runs. Mass spectrometry acquisition, data analysis, and elaboration were performed as described (34–36). Experimental design illustrations were generated using Biorender (www.biorender.com).

### Lipidomics

Total lipids were extracted as previously described (36): 10 μL of red blood cells (RBCs) were mixed with 90 μL of cold methanol. Samples were then briefly vortexed and incubated at −20°C for 30 min. Following incubation, samples were centrifuged at 18,000x*g* for 10 min at 4°C and 80 μL of supernatant was transferred to a fresh tube for analysis. Lipid extracts were analyzed (10 μL per injection) on a Thermo Vanquish UHPLC/Q Exactive MS system using a 5 min lipidomics gradient and a Kinetex C18 column (30 × 2.1 mm, 1.7 µm, Phenomenex) held at 50°C. Mobile phase A: 25:75 MeCN:water with 5 mM ammonium acetate; mobile phase B: 90:10 isopropanol:MeCN with 5 mM ammonium acetate. The gradient and flow rate were as follows: 0.3 mL/min of 10% B at 0 min, 0.3 mL/min of 95% B at 3 min, 0.3 mL/min of 95% B at 4.2 min, 0.45 mL/min 10% B at 4.3 min, 0.4 mL/min of 10% B at 4.9 min, and 0.3 mL/min of 10% B at 5 min. Samples were run in positive and negative ion modes (both electrospray ionization [ESI], separate runs) at 125 to 1,500 *m/z* and 70,000 resolution, 4 kV spray voltage, 45 sheath gas, and 25 auxiliary gas. The MS was run in data-dependent acquisition mode [ddMS (2)] with top 10 fragmentation. Raw MS data files were searched using LipidSearch v 5.0 (Thermo).

### Statistical analyses of omics data

Statistical analyses—including HCA, linear discriminant analysis (LDA), repeated measures ANOVA (including false discovery rate-correction), multivariate empirical Bayes statistical time-series analysis (MEBA; to track changes in lipids over time), and correlation analyses (Spearman correlation; to assess the degree of association between gonadotropins and metabolomic output) were performed using both MetaboAnalyst 5.0 (37) and in-house developed code in R (4.2.3 2023-03-15).

### Sample size, data analysis, and statistics

Published comparative studies of women with obesity compared with normal-weight women indicate a 50% reduction in LH pulse amplitude in women with obesity compared with those of normal weight (11). A sample size of 10 women would be sufficient to demonstrate changes in LH pulsatility given these estimates; however, as we were studying normal weight women given a high-fat diet, which may or may not mimic the conditions present in obesity, we aimed for a sample size of 20 women. This sample size was estimated to be able to detect a decrement of 25% in LH pulse amplitude (primary outcome) and was felt to provide sufficient power for secondary outcomes (mean LH and FSH and response to GnRH). The study was not powered to detect metabolomic or lipidomic changes (the latter was performed to verify adherence to the high-fat diet), as these were exploratory, hypothesis-generating goals of the parent experiment.

The frequency and amplitude of LH pulses were assessed using a modified Santen and Bardin method as described previously (20). This method considers a nadir to peak amplitude change of at least 20% to define a pulse. Analysis was also performed with an additional requirement that each LH pulse have at least two consecutive data points that met this criterion. Results using a single-point criterion yielded LH pulse frequency estimates consistent with the normal early follicular phase of the menstrual cycle and, therefore, this method's results are reported. All observations were paired to reduce interparticipant variation. Baseline characteristics are expressed as mean ± SD. When possible, data were transformed logarithmically to approximate a normal distribution and parametric testing was performed using paired t tests. When data could not be normalized, Wilcoxon signed rank tests were used to test for differences between the baseline and end-of-diet assessments.

## Supplementary Material

pgad440_Supplementary_Data

## Data Availability

Data for this study are available on clinicaltrials.gov (NCT02653092) and de-identified hormone data are available at Figshare (doi: 10.6084/m9.figshare.24777324). Metabolomics data are provided in Table S1. Raw mass spec files are available at https://www.metabolomicsworkbench.org/.
