# Supplementary material for: A high-fat eucaloric diet induces reprometabolic syndrome of obesity in normal weight women
Source: PNAS Nexus. 2023 Dec 18;3(1):pgad440. doi: 10.1093/pnasnexus/pgad440 (PMC10766410; doi:10.1093/pnasnexus/pgad440)
Supplement: pgad440_Supplementary_Data [file pgad440_supplementary_data.pdf]

PNAS nexus

**Supplementary Information for**

# A High-Fat Eucaloric Diet Induces Reprometabolic Syndrome of Obesity in Normal Weight Women

Nanette Santoro, MD[1], Katherine Kuhn, MS[1], Shannon Pretzel, BA[1], Irene E Schauer, MD, PhD[2], Angela Fought, MS[3], Angelo D'Alessandro, PhD[4], Daniel Stephenson, PhD[4], Andrew P Bradford, PhD[1]

Corresponding Author: Nanette Santoro, MD
Email: Nanette.santoro@cuanschutz.edu

**This PDF file includes:**

Figures S1 and S2

Data table S1

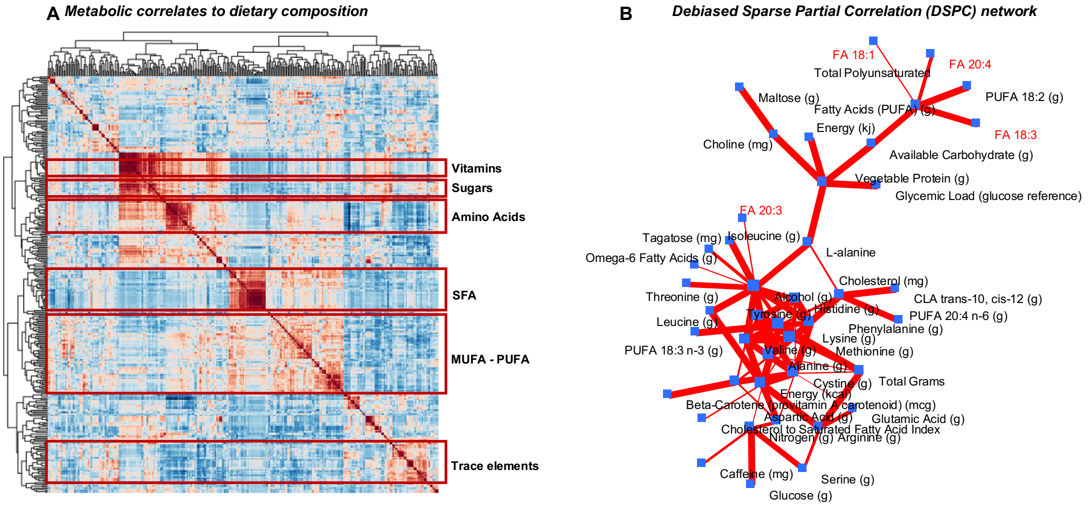


**Fig. S1.** (A) Metabolic correlates to dietary composition. A strong correlation is observed between dietary intake parameters (as defined by the components of each individual's diet) and small molecule metabolite levels in plasma. (B) Debiased Sparse Partial Correlation (DSPC) network analysis confirms the strong correlation between circulating fatty acids (red type) and amino acids.

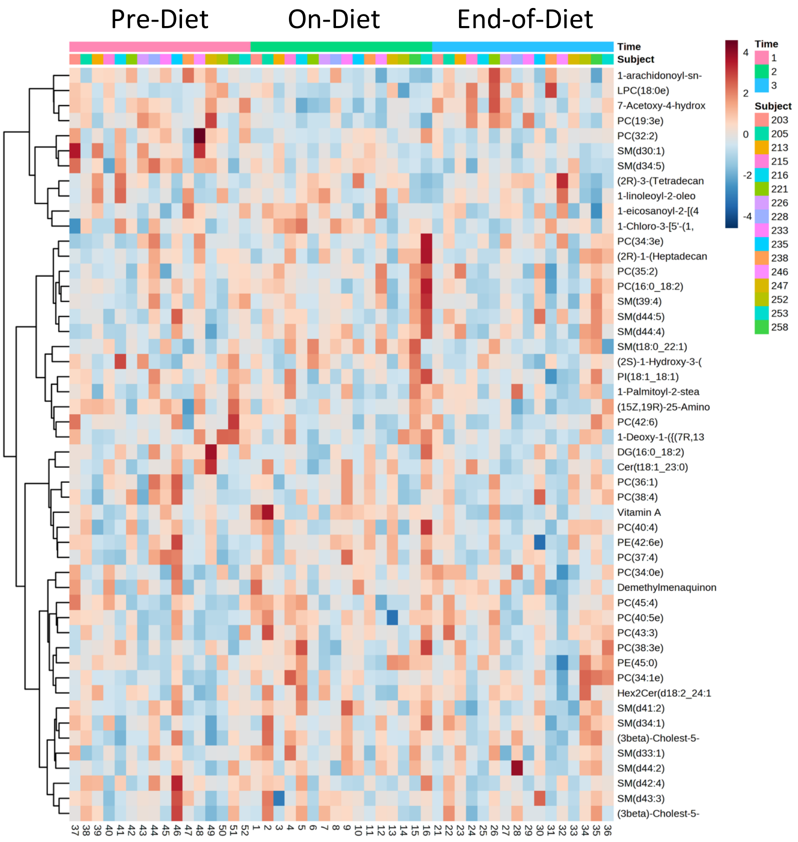


**Fig. S2.** Lipidomics analyses. Significant trends are observed for phosphatidylcholine and sphingomyelin, which increased during the HFD exposure, though trends are not as clear as for small molecule metabolites.

Data Table S1

| Sample | Compound | Participant II | stage 1 SC G | stage 2 SC G | stage 2 GIR/ | delta stage 2 |
|---|---|---|---|---|---|---|
| 40 | 01-Pre-diet | 215 | 0.8 | 10.4 | 0.21971831 | |
| 49 | 01-Pre-diet | 247 | 1.4 | 6.7 | 0.1415493 | |
| 42 | 01-Pre-diet | 221 | 1.6 | 9 | 0.22131148 | |
| 50 | 01-Pre-diet | 252 | 1.6 | 8 | 0.25 | |
| 43 | 01-Pre-diet | 226 | 1.7 | 12 | 0.35643564 | |
| 37 | 01-Pre-diet | 203 | 2 | 6.2 | 0.20217391 | |
| 45 | 01-Pre-diet | 233 | 2.2 | 12.8 | 0.31735537 | |
| 41 | 01-Pre-diet | 216 | 2.6 | 11.7 | 0.21272727 | |
| 44 | 01-Pre-diet | 228 | 2.6 | 10 | 0.18404908 | |
| 38 | 01-Pre-diet | 205 | 3.1 | 13.3 | 0.44333333 | |
| 46 | 01-Pre-diet | 235 | 3.1 | 11.3 | 0.18423913 | |
| 48 | 01-Pre-diet | 246 | 3.4 | 14.3 | 0.30642857 | |
| 52 | 01-Pre-diet | 253 | 3.6 | 13.4 | 0.31904762 | |
| 51 | 01-Pre-diet | 258 | 3.7 | 16.2 | 0.33061225 | |
| 39 | 01-Pre-diet | 213 | 4.5 | 16.2 | 0.47184466 | |
| 33 | 03-End of-die | 247 | 0.2 | 5.6 | 0.10306749 | 0.0385 |
| 29 | 03-End of-die | 233 | 1.1 | 6 | 0.27272727 | 0.0446 |
| 24 | 03-End of-die | 215 | 1.5 | 6.5 | 0.14028777 | 0.0794 |
| 27 | 03-End of-die | 226 | 1.6 | 9.8 | 0.3 | 0.0564 |
| 26 | 03-End of-die | 221 | 1.8 | 9.7 | 0.19144737 | 0.0299 |
| 36 | 03-End of-die | 253 | 1.8 | 10.3 | 0.28878505 | 0.0303 |
| 28 | 03-End of-die | 228 | 1.9 | 7.1 | 0.12383721 | 0.0602 |
| 32 | 03-End of-die | 246 | 2 | 14 | 0.25149701 | 0.0549 |
| 25 | 03-End of-die | 216 | 2.4 | 11.9 | 0.22884615 | -0.0161 |
| 34 | 03-End of-die | 252 | 2.5 | 9 | 0.23076923 | 0.0192 |
| 35 | 03-End of-die | 258 | 3 | 11.4 | 0.28983051 | 0.0408 |
| 22 | 03-End of-die | 205 | 3.3 | 12.2 | 0.24563758 | 0.1977 |
| 23 | 03-End of-die | 213 | 3.4 | 15.4 | 0.31006711 | 0.1618 |
| 30 | 03-End of-die | 235 | 4.1 | 12.6 | 0.16725664 | 0.017 |
| 21 | 03-End of-die | 203 | 4.4 | 16.5 | 0.28947368 | -0.2894737 |

| LH | FSH | Total Grams | Energy (kcal) | Total Fat (g) | Total Carboh | Total Protein |
|---|---|---|---|---|---|---|
| 8.04571429 | 8.4625 | | | | | |
| 1.64428571 | 5.16238095 | | | | | |
| 5.64190476 | 5.77380952 | | | | | |
| 3.49095238 | 3.41714286 | | | | | |
| 5.43142857 | 16.1866667 | | | | | |
| 5.46619048 | 12.0952381 | | | | | |
| 4.59666667 | 9.22333333 | | | | | |
| 4.52666667 | 7.62761905 | | | | | |
| 3.62095238 | 10.5909524 | | | | | |
| 6.58904762 | 8.98857143 | | | | | |
| 5.33571429 | 8.9855 | | | | | |
| 3.20571429 | 9.44476191 | | | | | |
| 6.24571429 | 7.31238095 | | | | | |
| 4.61571429 | 8.36380952 | | | | | |
| 4.40952381 | 6.44761905 | | | | | |
| 3.02 | 4.21047619 | 1521.8 | 2055.659 | 104.72 | 177.32 | 106.53 |
| 3.793 | 10.8119048 | 1569 | 2174.553 | 122.045 | 178.591 | 111.76 |
| 8.58809524 | 8.28619048 | 1670.745 | 2102.412 | 120.869 | 179.94 | 93.355 |
| 5.93809524 | 14.892381 | 1274 | 1719.563 | 91.565 | 149.667 | 81.939 |
| 5.55809524 | 11.1971429 | 1042.5 | 1919.393 | 100.063 | 164.029 | 92.074 |
| 5.73761905 | 5.724 | 1081.5 | 2134.179 | 112.798 | 182.609 | 103.719 |
| 3.64095238 | 6.61476191 | 1217 | 1742.671 | 92.521 | 155.694 | 83.477 |
| 3.62904762 | 9.11285714 | 1460.907 | 1790.118 | 96.465 | 152.338 | 91.99 |
| 4.64761905 | 6.10809524 | 1202.9 | 2237.609 | 115.724 | 190.946 | 112.809 |
| 2.71333333 | 3.88571429 | 1514.45 | 2054.054 | 101.251 | 178.229 | 110.909 |
| 4.43714286 | 5.96190476 | 966.217 | 2068.06 | 112.8 | 167.863 | 103.168 |
| 4.36333333 | 5.93380952 | 882.7 | 1530.011 | 80.556 | 140.992 | 63.497 |
| 2.60285714 | 11.5952381 | 1166.8 | 1783.977 | 96.663 | 151.699 | 86.843 |
| 2.64421053 | 7.33736842 | 1339.5 | 1671.483 | 91.787 | 132.937 | 86.475 |
| 3.86619048 | 11.8080952 | 1214.8 | 1801.287 | 103.385 | 139.258 | 87.227 |

| Animal Prote | Vegetable Pr | Alcohol (g) | Cholesterol ( | Total Saturat | Total Monou | Total Polyuns |
|---|---|---|---|---|---|---|
| 76.529 | 30 | 0 | 558.508 | 37.264 | 40.841 | 15.96 |
| 63.138 | 48.624 | 0.12 | 326.071 | 33.745 | 50.536 | 27.371 |
| 51.342 | 42.013 | 0.001 | 478.316 | 30.296 | 55.811 | 26.179 |
| 53.493 | 28.446 | 0.054 | 539.554 | 25.645 | 38.344 | 18.518 |
| 64.39 | 27.684 | 0.032 | 481.066 | 30.55 | 38.158 | 22.839 |
| 64.978 | 38.742 | 0.12 | 380.694 | 29.488 | 51.734 | 21.714 |
| 54.904 | 28.573 | 0 | 387.57 | 24.779 | 40.937 | 18.007 |
| 54.744 | 37.246 | 0.001 | 176.778 | 26.618 | 41.033 | 22.043 |
| 93.846 | 18.963 | 0 | 340.155 | 39.799 | 42.611 | 25.258 |
| 85.232 | 25.677 | 0 | 656.868 | 31.694 | 40.301 | 19.481 |
| 78.208 | 24.961 | 0 | 204.317 | 25.859 | 51.457 | 28.183 |
| 44.2 | 19.299 | 0 | 492.192 | 27.007 | 25.503 | 20.805 |
| 51.611 | 35.232 | 0 | 530.477 | 32.925 | 34.99 | 18.53 |
| 59.725 | 26.75 | 0 | 432.221 | 27.906 | 41.774 | 15.253 |
| 61.578 | 25.649 | 0 | 591.582 | 42.721 | 37.91 | 12.062 |

| Fructose (g) | Galactose (g | Glucose (g) | Lactose (g) | Maltose (g) | Sucrose (g) | Starch (g) |
|---|---|---|---|---|---|---|
| 10.19 | 0.101 | 11.347 | 3.612 | 2.538 | 37.162 | 80.283 |
| 15.641 | 1.099 | 11.601 | 3.59 | 0.305 | 30.643 | 68.671 |
| 29.447 | 0.345 | 26.908 | 1.711 | 0.906 | 15.999 | 61.928 |
| 6.604 | 1.062 | 7.551 | 5.907 | 1.827 | 25.008 | 71.821 |
| 4.044 | 0.321 | 4.877 | 7.886 | 2.005 | 4.133 | 119.113 |
| 7.858 | 0.121 | 9.205 | 1.164 | 2.016 | 28.028 | 101.441 |
| 12.055 | 0.164 | 13.238 | 1.714 | 1.959 | 16.7 | 74.697 |
| 9.874 | 0.372 | 8.805 | 0.321 | 0.263 | 23.102 | 77.688 |
| 10.887 | 0.241 | 18.996 | 4.111 | 4.268 | 26.637 | 91.593 |
| 3.375 | 0.214 | 4.822 | 4.295 | 1.758 | 23.721 | 111.61 |
| 12.067 | 0.52 | 12.341 | 8.418 | 1.917 | 36.127 | 68.353 |
| 7.765 | 0.22 | 7.982 | 1.795 | 1.82 | 17.477 | 81.662 |
| 5.883 | 0.107 | 5.852 | 2.097 | 0.91 | 7.236 | 87.568 |
| 2.026 | 0.419 | 3.124 | 13.171 | 0.456 | 28.317 | 58.94 |
| 9.652 | 0.067 | 6.069 | 4.703 | 0.44 | 27.76 | 64.901 |

| Total Dietary | Soluble Dieta | Insoluble Die | Pectins (g) | Total Vitami | Beta-Caroter | Retinol (mcg |
|---|---|---|---|---|---|---|
| 24.115 | 6.593 | 16.503 | 3.291 | 4685.68 | 2010.611 | 400.487 |
| 26.963 | 5.876 | 21.091 | 5.09 | 2254.728 | 960.826 | 196.024 |
| 33.286 | 4.963 | 28.258 | 5.792 | 11725.103 | 6408.514 | 312.625 |
| 16.136 | 5.223 | 10.87 | 2.697 | 2374.736 | 661.157 | 381.598 |
| 13.895 | 2.827 | 11.079 | 1.513 | 7550.137 | 3584.15 | 473.126 |
| 15.767 | 2.642 | 13.04 | 2.217 | 5572.37 | 3072.6 | 135.64 |
| 16.984 | 4.916 | 12.071 | 2.529 | 10183.262 | 4944.269 | 582.54 |
| 25.901 | 5.359 | 20.32 | 3.916 | 7782.875 | 3525.519 | 572.231 |
| 12.338 | 4.16 | 8.181 | 0.551 | 3690.563 | 687.339 | 763.84 |
| 17.926 | 4.989 | 12.931 | 2.019 | 4985.218 | 2156.219 | 417.122 |
| 17.975 | 3.842 | 14.114 | 3.655 | 8325.106 | 4423.427 | 286.089 |
| 12.915 | 4.097 | 8.839 | 1.931 | 8442.219 | 4220.775 | 421.737 |
| 26.204 | 4.614 | 21.609 | 2.83 | 2598.651 | 456.317 | 551.053 |
| 17.218 | 4.135 | 12.931 | 2.754 | 15699.339 | 8735.339 | 342.374 |
| 16.726 | 4.176 | 12.762 | 2.632 | 3308.407 | 931.195 | 527.367 |

| Vitamin D (c | Total Alpha-T | Vitamin E (T | Beta-Tocoph | Gamma-Toc | Delta-Tocoph | Vitamin K (p |
|---|---|---|---|---|---|---|
| 2.401 | 18.368 | 16.973 | 0.247 | 12.898 | 0.698 | 72.474 |
| 1.451 | 22.915 | 21.203 | 0.56 | 14.808 | 0.857 | 117.801 |
| 3.153 | 25.155 | 22.821 | 0.562 | 20.856 | 2.348 | 229.109 |
| 3.158 | 13.064 | 11.741 | 0.174 | 12.435 | 1.018 | 37.169 |
| 2.737 | 12.02 | 9.822 | 0.283 | 20.483 | 3.667 | 132.852 |
| 2.119 | 14.074 | 12.063 | 0.438 | 18.127 | 2.379 | 76.964 |
| 1.828 | 16.605 | 15.193 | 0.263 | 12.818 | 2.497 | 166.416 |
| 2.605 | 20.509 | 18.478 | 0.431 | 17.968 | 6.26 | 246.462 |
| 29.079 | 11.614 | 9.213 | 0.411 | 21.835 | 5.261 | 76.609 |
| 3.076 | 14.192 | 12.685 | 0.466 | 12.998 | 2.089 | 137.442 |
| 17.059 | 22.556 | 20.251 | 2.161 | 14.239 | 1.652 | 44.942 |
| 3.13 | 10.954 | 9.107 | 0.485 | 16.164 | 3.707 | 126.846 |
| 4.014 | 16.241 | 14.953 | 0.514 | 10.597 | 2.32 | 51.326 |
| 2.036 | 16.277 | 15.14 | 0.184 | 10.542 | 0.938 | 109.013 |
| 3.634 | 10.958 | 9.949 | 0.572 | 7.679 | 1.316 | 130.396 |

| Vitamin C (a: | Thiamin (vita | Riboflavin (vi | Niacin (vitam | Pantothenic / | Vitamin B-6 | Total Folate |
|---|---|---|---|---|---|---|
| 116.215 | 2.243 | 1.894 | 28.177 | 5.563 | 1.942 | 300.704 |
| 203.585 | 0.876 | 1.703 | 26.443 | 4.684 | 1.841 | 305.699 |
| 122.789 | 1.4 | 1.849 | 26.907 | 4.915 | 2.15 | 500.639 |
| 133.744 | 1.222 | 2.127 | 14.042 | 4.834 | 1.138 | 306.092 |
| 92.69 | 1.468 | 1.902 | 21.967 | 4.548 | 1.262 | 340.289 |
| 74.788 | 1.641 | 1.523 | 27.654 | 4.342 | 1.942 | 321.788 |
| 89.696 | 1.373 | 2.048 | 23.772 | 3.573 | 1.604 | 361.628 |
| 125.92 | 1.57 | 2.321 | 33.942 | 3.53 | 2.521 | 719.355 |
| 12.591 | 1.171 | 1.645 | 30.13 | 5.413 | 2.1 | 184.851 |
| 70.622 | 1.531 | 2.344 | 24.807 | 6.192 | 2.253 | 356.429 |
| 37.651 | 1.091 | 1.327 | 28.38 | 5.181 | 2.053 | 222.707 |
| 80.715 | 1.342 | 1.442 | 13.938 | 4.06 | 1.395 | 336.074 |
| 94.441 | 1.111 | 2.001 | 19.389 | 4.791 | 1.252 | 334.202 |
| 40.985 | 1.092 | 1.946 | 16.995 | 5.15 | 1.236 | 293.505 |
| 71.892 | 1.02 | 1.521 | 21.818 | 4.852 | 1.821 | 246.736 |

| Vitamin B-12 | Calcium (mg | Phosphorus ( | Magnesium ( | Iron (mg) | Zinc (mg) | Copper (mg) |
|---|---|---|---|---|---|---|
| 3.622 | 856.855 | 1311.821 | 275.021 | 16.212 | 12.445 | 1.313 |
| 3.12 | 757.92 | 1968.256 | 632.452 | 13.898 | 14.273 | 1.807 |
| 2.862 | 713.831 | 1413.015 | 422.176 | 12.793 | 12.93 | 1.508 |
| 4.258 | 781.811 | 1388.708 | 323.267 | 11.174 | 11.003 | 1.137 |
| 3.223 | 1125.229 | 1442.576 | 255.439 | 10.611 | 10.23 | 1.037 |
| 4.716 | 530.971 | 1300.55 | 335.157 | 16.732 | 18.341 | 1.743 |
| 3.697 | 1040.131 | 1379.01 | 315.882 | 14.841 | 10.482 | 1.43 |
| 4.036 | 975.222 | 1428.066 | 406.891 | 18.943 | 15.774 | 1.382 |
| 8.251 | 828.092 | 1553.964 | 218.548 | 13.066 | 13.283 | 0.796 |
| 5.725 | 1119.637 | 1651.921 | 296.4 | 14.324 | 15.015 | 1.086 |
| 4.697 | 840.507 | 1384.802 | 368.609 | 8.986 | 8.162 | 1.705 |
| 2.938 | 786.408 | 987.159 | 170.173 | 9.387 | 8.297 | 0.776 |
| 4.461 | 1113.24 | 1522.938 | 396.438 | 12.78 | 11.436 | 1.569 |
| 3.626 | 892.235 | 1406.126 | 315.421 | 10.086 | 11.241 | 1.424 |
| 3.598 | 926.715 | 1224.64 | 262.516 | 9.339 | 11.972 | 0.913 |

| Selenium (m | Sodium (mg) | Potassium (r | SFA 4:0 (buty | SFA 6:0 (capr | SFA 8:0 (capr | SFA 10:0 (ca |
|---|---|---|---|---|---|---|
| 113.319 | 2620.597 | 2495.31 | 0.729 | 0.534 | 0.618 | 0.98 |
| 97.455 | 2154.64 | 2626.302 | 0.223 | 0.258 | 1.01 | 1.019 |
| 96.309 | 1544.252 | 3702.786 | 0.559 | 0.364 | 0.229 | 0.557 |
| 125.899 | 1633.705 | 2162.799 | 0.566 | 0.417 | 0.26 | 0.599 |
| 163.157 | 2915.239 | 1367.662 | 0.748 | 0.583 | 0.392 | 0.909 |
| 152.906 | 4141.054 | 2207.102 | 0.187 | 0.147 | 0.162 | 0.469 |
| 114.955 | 2403.091 | 2123.735 | 0.41 | 0.347 | 0.247 | 0.556 |
| 77.906 | 1306.946 | 2332.627 | 0.614 | 0.325 | 0.603 | 0.832 |
| 128.057 | 3067.965 | 1759.959 | 1.169 | 0.596 | 0.417 | 0.877 |
| 155.6 | 3773.06 | 2946.343 | 0.45 | 0.379 | 0.255 | 0.642 |
| 104.117 | 2157.061 | 2519.466 | 0.413 | 0.287 | 0.299 | 0.532 |
| 106.099 | 2784.659 | 1980.009 | 0.724 | 0.524 | 0.327 | 0.756 |
| 175.169 | 1902.007 | 1964.08 | 0.801 | 0.689 | 0.466 | 1.066 |
| 103.809 | 1663.637 | 1977.972 | 0.674 | 0.492 | 0.636 | 0.939 |
| 119.259 | 1616.698 | 2163.31 | 1.145 | 0.823 | 0.505 | 1.179 |

| SFA 12:0 (lau | SFA 14:0 (my | SFA 16:0 (pa | SFA 17:0 (ma | SFA 18:0 (ste | SFA 20:0 (ara | SFA 22:0 (be |
|---|---|---|---|---|---|---|
| 2.719 | 3.707 | 17.869 | 0.3 | 8.983 | 0.334 | 0.132 |
| 5.795 | 3.472 | 14.221 | 0.126 | 5.59 | 0.534 | 0.887 |
| 0.562 | 2.442 | 16.544 | 0.132 | 6.851 | 0.577 | 0.905 |
| 0.663 | 2.227 | 13.89 | 0.241 | 5.966 | 0.308 | 0.235 |
| 1.181 | 3.25 | 15.964 | 0.22 | 6.488 | 0.278 | 0.157 |
| 0.62 | 1.823 | 16.488 | 0.185 | 7.628 | 0.426 | 0.755 |
| 0.791 | 2.201 | 13.529 | 0.194 | 5.87 | 0.171 | 0.102 |
| 2.776 | 3.502 | 12.084 | 0.079 | 4.512 | 0.206 | 0.105 |
| 0.895 | 4.293 | 20.422 | 0.307 | 9.339 | 0.292 | 0.156 |
| 0.674 | 2.959 | 17.499 | 0.255 | 7.82 | 0.229 | 0.111 |
| 1.939 | 2.663 | 14.004 | 0.133 | 5.046 | 0.263 | 0.095 |
| 0.841 | 2.645 | 14.86 | 0.215 | 5.716 | 0.179 | 0.106 |
| 1.311 | 3.859 | 17.17 | 0.28 | 6.688 | 0.118 | 0.072 |
| 2.903 | 3.191 | 12.922 | 0.21 | 5.432 | 0.186 | 0.082 |
| 1.267 | 4.38 | 22.093 | 0.317 | 9.695 | 0.365 | 0.638 |

| MUFA 14:1 ( | MUFA 16:1 ( | MUFA 18:1 ( | MUFA 20:1 ( | MUFA 22:1 ( | PUFA 18:2 (li | PUFA 18:3 (li |
|---|---|---|---|---|---|---|
| 0.287 | 1.618 | 38.054 | 0.606 | 0.005 | 12.132 | 3.27 |
| 0.154 | 0.821 | 48.761 | 0.501 | 0.024 | 25.504 | 1.406 |
| 0.194 | 1.381 | 52.997 | 0.753 | 0.044 | 21.906 | 3.791 |
| 0.188 | 0.962 | 36.781 | 0.458 | 0.01 | 15.696 | 2.447 |
| 0.273 | 1.078 | 36.217 | 0.493 | 0.003 | 18.681 | 3.703 |
| 0.321 | 1.594 | 48.719 | 0.552 | 0.02 | 19.341 | 2.049 |
| 0.224 | 0.978 | 39.394 | 0.324 | 0.004 | 15.802 | 1.796 |
| 0.112 | 0.958 | 39.255 | 0.42 | 0.022 | 18.373 | 3.314 |
| 0.181 | 2.542 | 37.651 | 1.105 | 0.765 | 19.424 | 3.254 |
| 0.241 | 1.391 | 38.08 | 0.4 | 0.005 | 16.587 | 2.323 |
| 0.065 | 1.557 | 47.718 | 2.024 | 0 | 20.811 | 2.535 |
| 0.185 | 0.975 | 23.962 | 0.24 | 0.003 | 18.112 | 2.397 |
| 0.32 | 1.156 | 33.168 | 0.182 | 0.006 | 16.389 | 1.439 |
| 0.151 | 1.115 | 39.921 | 0.393 | 0.003 | 12.597 | 2.336 |
| 0.34 | 1.878 | 34.797 | 0.392 | 0.02 | 10.695 | 0.862 |

| PUFA 18:4 (p | PUFA 20:4 (a | PUFA 20:5 n- | PUFA 22:5 n- | PUFA 22:6 n- | Tryptophan ( | Threonine (g |
|---|---|---|---|---|---|---|
| 0 | 0.289 | 0.024 | 0.037 | 0.074 | 1.245 | 4.262 |
| 0 | 0.209 | 0.013 | 0.026 | 0.037 | 1.449 | 4.301 |
| 0 | 0.203 | 0.009 | 0.009 | 0.042 | 0.97 | 3.367 |
| 0 | 0.239 | 0.009 | 0.008 | 0.044 | 1.108 | 3.304 |
| 0 | 0.237 | 0.021 | 0.032 | 0.057 | 1.398 | 3.848 |
| 0 | 0.164 | 0.003 | 0.002 | 0.019 | 0.95 | 3.902 |
| 0.001 | 0.199 | 0.023 | 0.025 | 0.049 | 1.045 | 3.249 |
| 0 | 0.121 | 0.016 | 0.031 | 0.038 | 1.192 | 3.526 |
| 0.137 | 0.292 | 0.496 | 0.307 | 1.05 | 1.253 | 4.41 |
| 0 | 0.299 | 0.014 | 0.028 | 0.049 | 1.418 | 4.791 |
| 0.221 | 1.62 | 0.84 | 0.228 | 1.779 | 1.36 | 4.29 |
| 0 | 0.194 | 0.012 | 0.013 | 0.038 | 0.899 | 2.551 |
| 0.002 | 0.234 | 0.04 | 0.022 | 0.206 | 1.225 | 3.3 |
| 0 | 0.187 | 0.013 | 0.021 | 0.046 | 1.043 | 3.558 |
| 0 | 0.256 | 0.012 | 0.016 | 0.042 | 0.979 | 3.303 |

| Isoleucine (g | Leucine (g) | Lysine (g) | Methionine ( | Cystine (g) | Phenylalanin | Tyrosine (g) |
|---|---|---|---|---|---|---|
| 5.056 | 8.06 | 7.216 | 2.499 | 1.389 | 4.664 | 3.529 |
| 4.913 | 8.862 | 6.696 | 2.325 | 1.467 | 5.137 | 3.806 |
| 3.905 | 6.774 | 5.537 | 1.91 | 1.147 | 4.136 | 3.07 |
| 3.708 | 6.597 | 4.945 | 1.756 | 1.174 | 3.82 | 2.856 |
| 4.53 | 7.256 | 5.419 | 2.126 | 1.215 | 4.225 | 3.375 |
| 4.486 | 8.021 | 6.746 | 2.243 | 1.428 | 4.599 | 3.352 |
| 3.873 | 6.395 | 4.982 | 1.841 | 1.107 | 3.836 | 2.84 |
| 4.452 | 7.017 | 6.002 | 1.964 | 1.138 | 4.139 | 3.172 |
| 5.271 | 8.748 | 8.239 | 2.822 | 1.401 | 4.712 | 3.791 |
| 5.277 | 9.326 | 8.04 | 2.732 | 1.358 | 5.18 | 4.316 |
| 4.932 | 8.222 | 7.684 | 2.447 | 1.31 | 4.393 | 3.412 |
| 2.806 | 4.763 | 3.725 | 1.44 | 0.843 | 2.799 | 2.313 |
| 3.803 | 6.531 | 4.609 | 1.82 | 1.073 | 3.901 | 2.955 |
| 4.138 | 6.907 | 5.492 | 1.975 | 1.088 | 4.008 | 3.156 |
| 3.809 | 6.546 | 5.521 | 1.966 | 1.134 | 3.797 | 3.044 |

| Valine (g) | Arginine (g) | Histidine (g) | Alanine (g) | Aspartic Acic | Glutamic Aci | Glycine (g) |
|---|---|---|---|---|---|---|
| 5.419 | 5.907 | 2.893 | 5.303 | 9.7 | 19.207 | 4.871 |
| 5.521 | 8.691 | 2.96 | 5.548 | 11.288 | 20.656 | 5.307 |
| 4.469 | 5.823 | 2.402 | 4.364 | 9.158 | 18.454 | 4.088 |
| 4.235 | 4.847 | 2.05 | 3.683 | 7.606 | 16.97 | 3.188 |
| 5.009 | 4.357 | 2.381 | 4.065 | 7.462 | 18.595 | 3.35 |
| 5.196 | 6.881 | 2.89 | 5.338 | 9.386 | 20.444 | 5.33 |
| 4.278 | 4.787 | 2.218 | 3.889 | 7.274 | 17.728 | 3.575 |
| 4.714 | 5.651 | 2.551 | 4.289 | 8.555 | 17.554 | 3.999 |
| 5.742 | 6.163 | 3.228 | 5.769 | 9.437 | 21.039 | 5.523 |
| 6.048 | 6.295 | 3.386 | 5.611 | 10.253 | 21.491 | 4.476 |
| 5.371 | 6.06 | 2.835 | 5.215 | 10.012 | 18.778 | 4.408 |
| 3.184 | 2.904 | 1.616 | 2.763 | 5.658 | 11.895 | 2.175 |
| 4.491 | 4.485 | 2.039 | 3.754 | 7.682 | 16.369 | 3.175 |
| 4.765 | 4.953 | 2.255 | 4.005 | 7.579 | 16.576 | 3.452 |
| 4.339 | 5.074 | 2.351 | 4.16 | 8.024 | 15.166 | 3.855 |

| Proline (g) | Serine (g) | Aspartame ( | Saccharin (m | Caffeine (mg | Phytic Acid (r | Oxalic Acid ( |
|---|---|---|---|---|---|---|
| 6.111 | 4.631 | 0 | 0 | 101.426 | 916.813 | 154.801 |
| 6.15 | 5.297 | 0 | 0 | 3.03 | 1860.862 | 344.334 |
| 5.075 | 4.388 | 0 | 0 | 0 | 918.693 | 430.242 |
| 5.583 | 4.184 | 0 | 0 | 94.8 | 872.452 | 168.634 |
| 6.773 | 4.048 | 0 | 0 | 0 | 666.806 | 126.455 |
| 6.319 | 4.973 | 0 | 0 | 0 | 1252.122 | 216.553 |
| 5.579 | 3.735 | 12.5 | 0 | 2.37 | 748.119 | 401.506 |
| 5.281 | 3.883 | 0 | 0 | 47.4 | 1348.701 | 452.613 |
| 7.083 | 4.676 | 0 | 0 | 98.44 | 327.348 | 82.365 |
| 7.037 | 5.399 | 0 | 0 | 96.68 | 809.431 | 345.823 |
| 5.383 | 4.339 | 0 | 0 | 5.027 | 939.237 | 335.09 |
| 4.141 | 2.881 | 0 | 0 | 0.66 | 276.065 | 94.151 |
| 5.419 | 3.884 | 0 | 0 | 94.8 | 1182.803 | 319.384 |
| 5.817 | 4.02 | 0 | 0 | 94.8 | 854.455 | 255.188 |
| 5.299 | 4.057 | 0 | 0 | 4.35 | 643.071 | 171.416 |

| 3-Methylhist | Sucrose Poly | Ash (g) | Water (g) | % Calories fr | % Calories fr | % Calories fr |
|---|---|---|---|---|---|---|
| 24.312 | 0 | 16.368 | 1171.456 | 45.22 | 33.886 | 20.9 |
| 17.03 | 0 | 16.017 | 1140.565 | 48.332 | 32.122 | 19.483 |
| 14.168 | 0 | 14.59 | 1272.058 | 50.144 | 32.634 | 17.052 |
| 7.095 | 0 | 12.883 | 949.355 | 46.592 | 34.406 | 18.929 |
| 11.611 | 0 | 14.965 | 681.881 | 46.084 | 34.494 | 19.352 |
| 26.559 | 0 | 15.82 | 668.048 | 46.037 | 34.272 | 19.595 |
| 14.245 | 0 | 14.723 | 871.04 | 46.364 | 34.402 | 19.192 |
| 16.938 | 0 | 12.487 | 1109.791 | 46.771 | 33.218 | 19.901 |
| 45.021 | 0 | 15.303 | 771.897 | 46.005 | 32.972 | 20.975 |
| 26.013 | 0 | 17.185 | 1106.529 | 43.65 | 34.617 | 21.499 |
| 36.333 | 0 | 11.62 | 573.763 | 47.722 | 31.912 | 20.259 |
| 8.968 | 0 | 13.416 | 593.268 | 46.794 | 36.631 | 16.508 |
| 0 | 0 | 14.006 | 821.43 | 47.497 | 33.511 | 18.977 |
| 11.651 | 0 | 13.536 | 1015.832 | 47.833 | 31.53 | 20.632 |
| 18.227 | 0 | 11.673 | 882.822 | 50.62 | 30.037 | 19.382 |

| % Calories fr | % Calories fr | % Calories fr | % Calories fr | Polyunsatura | Cholesterol t | Total Vitami |
|---|---|---|---|---|---|---|
| 0 | 16.081 | 17.669 | 6.868 | 0.428 | 65.562 | 735.589 |
| 0.039 | 13.633 | 19.859 | 10.688 | 0.811 | 50.386 | 356.162 |
| 0 | 12.671 | 23.089 | 10.796 | 0.864 | 54.515 | 1380.711 |
| 0.022 | 13.157 | 19.484 | 9.33 | 0.722 | 52.879 | 491.791 |
| 0.012 | 14.145 | 17.521 | 10.477 | 0.748 | 54.909 | 1070.484 |
| 0.039 | 12.146 | 21.042 | 8.781 | 0.736 | 48.817 | 647.74 |
| 0 | 12.558 | 20.38 | 8.976 | 0.727 | 44.405 | 1406.585 |
| 0.001 | 12.973 | 19.872 | 10.647 | 0.828 | 35.723 | 1159.818 |
| 0 | 15.811 | 16.96 | 10.014 | 0.635 | 57.205 | 878.396 |
| 0 | 13.716 | 17.346 | 8.355 | 0.615 | 64.855 | 776.492 |
| 0 | 10.994 | 21.661 | 11.957 | 1.09 | 36.333 | 1023.326 |
| 0 | 15.711 | 14.826 | 12.038 | 0.77 | 51.887 | 1125.2 |
| 0 | 16.364 | 16.985 | 9.073 | 0.563 | 59.778 | 627.106 |
| 0 | 14.622 | 21.708 | 7.9 | 0.547 | 49.796 | 1798.264 |
| 0 | 20.995 | 18.525 | 5.83 | 0.282 | 72.728 | 682.566 |

| TRANS 18:1 ( | TRANS 18:2 ( | TRANS 16:1 ( | Total Trans-F | Beta-Caroter | Alpha-Carote | Beta-Cryptox |
|---|---|---|---|---|---|---|
| 1.398 | 0.289 | 0.076 | 1.852 | 1915.295 | 68.051 | 122.581 |
| 0.73 | 0.206 | 0.051 | 1.002 | 928.364 | 31.282 | 33.642 |
| 1.053 | 0.335 | 0.046 | 1.443 | 5980.235 | 665.087 | 191.47 |
| 0.851 | 0.221 | 0.046 | 1.119 | 616.357 | 63.498 | 26.102 |
| 0.961 | 0.378 | 0.088 | 1.443 | 2996.536 | 1107.723 | 67.504 |
| 1.549 | 0.225 | 0.066 | 1.851 | 2626.024 | 842.455 | 50.696 |
| 0.725 | 0.24 | 0.067 | 1.046 | 4868.351 | 130.884 | 20.951 |
| 0.585 | 0.287 | 0.045 | 0.948 | 3382.208 | 242.435 | 44.186 |
| 1.47 | 0.438 | 0.062 | 1.996 | 652.663 | 60.09 | 9.262 |
| 1.587 | 0.363 | 0.087 | 2.035 | 2082.449 | 58.075 | 89.464 |
| 0.984 | 0.228 | 0.043 | 1.275 | 63.329 | 0.653 | 3.802 |
| 1.069 | 0.342 | 0.058 | 1.469 | 4163.96 | 95.892 | 17.737 |
| 1.147 | 0.299 | 0.118 | 1.57 | 409.895 | 50.78 | 42.063 |
| 0.933 | 0.207 | 0.036 | 1.199 | 7307.196 | 2708.372 | 147.916 |
| 2.46 | 0.411 | 0.095 | 2.974 | 906.325 | 24.662 | 25.079 |

| Lutein + Zea) | Lycopene (m | Dietary Folat | Natural Fola | Synthetic Fol | Total Vitami | Energy (kj) |
|---|---|---|---|---|---|---|
| 1890.588 | 16715.071 | 352.724 | 225.984 | 74.72 | 568.038 | 8600.877 |
| 1676.27 | 1697.64 | 337.175 | 260.681 | 45.018 | 276.093 | 9098.328 |
| 6141.483 | 15926.901 | 520.019 | 473.099 | 27.54 | 846.668 | 8796.493 |
| 669.062 | 8935.755 | 399.119 | 173.073 | 133.019 | 436.694 | 7194.652 |
| 1435.504 | 0 | 451.889 | 180.99 | 159.299 | 771.805 | 8030.742 |
| 959.526 | 12997.463 | 433.882 | 161.703 | 160.085 | 391.69 | 8929.403 |
| 2641.442 | 9435.644 | 472.878 | 202.118 | 159.51 | 994.562 | 7291.334 |
| 3612.212 | 1749.714 | 998.665 | 320.675 | 398.68 | 866.024 | 7489.854 |
| 818.861 | 1312.23 | 229.221 | 121.135 | 63.716 | 821.118 | 9362.155 |
| 3015.794 | 5885.337 | 433.172 | 246.615 | 109.814 | 596.807 | 8594.163 |
| 223.205 | 0.038 | 181.005 | 126.757 | 31.775 | 146.451 | 8182.962 |
| 2190.089 | 2341.43 | 395.324 | 251.264 | 84.81 | 773.469 | 6401.564 |
| 803.292 | 1878.02 | 354.842 | 304.682 | 29.52 | 589.079 | 7464.161 |
| 2938.217 | 9767.305 | 341.2 | 225.445 | 68.06 | 1070.319 | 6993.487 |
| 1892.313 | 0 | 262.696 | 224.056 | 22.68 | 604.966 | 7536.583 |

| Niacin Equiva | Total Sugars | Omega-3 Fa | Manganese ( | Vitamin E (Ir | Natural Alph | Synthetic Alp |
|---|---|---|---|---|---|---|
| 48.93 | 65.129 | 3.365 | 3.487 | 25.311 | 16.973 | 0 |
| 50.595 | 62.494 | 1.422 | 5.72 | 31.658 | 21.203 | 0 |
| 43.07 | 75.33 | 3.771 | 5.443 | 34.063 | 22.821 | 0 |
| 32.504 | 47.935 | 2.483 | 3.019 | 17.526 | 11.741 | 0 |
| 45.259 | 23.205 | 3.758 | 3.149 | 14.652 | 9.822 | 0 |
| 43.489 | 48.463 | 2.057 | 3.279 | 18.005 | 12.063 | 0 |
| 41.193 | 45.819 | 1.876 | 2.314 | 22.679 | 15.193 | 0 |
| 53.817 | 42.339 | 3.395 | 6.088 | 27.576 | 18.478 | 0 |
| 51.02 | 65.221 | 5.07 | 2.26 | 13.737 | 9.213 | 0 |
| 48.445 | 38.254 | 2.391 | 3.272 | 18.917 | 12.685 | 0 |
| 40.638 | 75.449 | 2.942 | 4.909 | 18.632 | 12.481 | 0 |
| 28.927 | 36.839 | 2.3 | 2.332 | 13.597 | 9.107 | 0 |
| 39.802 | 22.062 | 1.625 | 5.692 | 22.307 | 14.953 | 0 |
| 34.384 | 47.676 | 2.319 | 2.838 | 22.586 | 15.14 | 0 |
| 38.128 | 48.625 | 0.692 | 3.225 | 14.846 | 9.949 | 0 |

| Daidzein (mg | Genistein (m | Glycitein (mg | Coumestrol ( | Biochanin A ( | Formononeti | Added Sugar |
|---|---|---|---|---|---|---|
| 0.084 | 0.821 | 0 | 0.059 | 0.048 | 0 | 29.694 |
| 0.133 | 0.062 | 0.021 | 0.003 | 0 | 0 | 25.633 |
| 0.029 | 0.053 | 0 | 0.005 | 0 | 0 | 16.817 |
| 0.092 | 0.038 | 0 | 0.059 | 0.001 | 0.001 | 21.863 |
| 0.2 | 0.15 | 0.006 | 0.001 | 0 | 0 | 6.205 |
| 0.276 | 0.192 | 0.021 | 0.003 | 1.862 | 0 | 32.531 |
| 0.019 | 0.013 | 0 | 0.005 | 0 | 0 | 22.127 |
| 15.498 | 15.843 | 1.89 | 0.009 | 0 | 0.002 | 27.123 |
| 0.645 | 0.82 | 0.129 | 0.059 | 0 | 0 | 74.435 |
| 0.095 | 0.06 | 0 | 0.059 | 0 | 0 | 20.44 |
| 0.003 | 0.004 | 0 | 0.004 | 0 | 0 | 63.927 |
| 0.08 | 0.104 | 0 | 0 | 0 | 0.001 | 25.579 |
| 0.107 | 0.09 | 0 | 0.066 | 0 | 0 | 10.224 |
| 2.345 | 2.498 | 0.311 | 0.059 | 0 | 0 | 19.447 |
| 0.182 | 0.291 | 0.043 | 0 | 0 | 0 | 26.147 |

| Acesulfame | Sucralose (m | Available Ca | Glycemic Ind | Glycemic Ind | Glycemic Loa | Glycemic Loa |
|---|---|---|---|---|---|---|
| 0 | 0 | 153.204 | 50.969 | 72.866 | 78.086 | 111.634 |
| 0 | 0 | 151.628 | 55.616 | 79.506 | 84.33 | 120.554 |
| 0 | 0 | 146.654 | 55.081 | 78.742 | 80.779 | 115.478 |
| 0 | 0 | 133.531 | 52.289 | 74.765 | 69.823 | 99.834 |
| 0 | 0 | 150.133 | 52.414 | 74.934 | 78.691 | 112.501 |
| 0 | 0 | 166.842 | 57.322 | 81.949 | 95.636 | 136.726 |
| 12.5 | 12.5 | 136.61 | 49.016 | 70.051 | 66.961 | 95.697 |
| 0 | 0 | 126.437 | 57.042 | 81.535 | 72.122 | 103.091 |
| 0 | 0 | 178.607 | 67.18 | 96.036 | 119.988 | 171.527 |
| 0 | 0 | 160.303 | 49.097 | 70.185 | 78.704 | 112.508 |
| 0 | 0 | 164.938 | 62.472 | 89.302 | 103.04 | 147.293 |
| 0 | 0 | 128.076 | 70.357 | 100.604 | 90.11 | 128.85 |
| 0 | 0 | 125.495 | 53.116 | 75.953 | 66.658 | 95.317 |
| 0 | 0 | 115.719 | 45.575 | 65.172 | 52.739 | 75.416 |
| 0 | 0 | 122.532 | 63.153 | 90.287 | 77.382 | 110.63 |

| Choline (mg) | Betaine (mg | Erythritol (g) | Inositol (g) | Isomalt (g) | Lactitol (g) | Maltitol (g) |
|---|---|---|---|---|---|---|
| 543.717 | 48.821 | 0 | 0.511 | 0 | 0 | 0 |
| 424.977 | 22.232 | 0 | 0.629 | 0 | 0 | 0 |
| 571.71 | 97.669 | 0 | 0.571 | 0 | 0 | 0 |
| 496.677 | 98.411 | 0 | 0.357 | 0 | 0 | 0 |
| 420.899 | 204.182 | 0 | 0.315 | 0 | 0 | 0 |
| 454.126 | 202.979 | 0 | 0.332 | 0 | 0 | 0 |
| 372.909 | 117.296 | 0.006 | 0.649 | 0 | 0 | 0 |
| 245.226 | 43.14 | 0 | 0.422 | 0 | 0 | 0 |
| 362.485 | 147.867 | 0 | 0.109 | 0 | 0 | 0 |
| 607.14 | 108.668 | 0 | 0.273 | 0 | 0 | 0 |
| 247.89 | 211.932 | 0.001 | 0.397 | 0 | 0 | 0 |
| 428.244 | 152.121 | 0 | 0.236 | 0 | 0 | 0 |
| 466.374 | 133.119 | 0 | 0.52 | 0 | 0 | 0 |
| 435.599 | 78.149 | 0 | 0.261 | 0 | 0 | 0 |
| 536.46 | 101.194 | 0 | 0.23 | 0 | 0 | 0 |

| Mannitol (g) | Pinitol (g) | Sorbitol (g) | Xylitol (g) | Nitrogen (g) | Total Conjug | CLA cis-9, tra |
|---|---|---|---|---|---|---|
| 0.067 | 0 | 0.07 | 0.04 | 17.079 | 0.204 | 0.166 |
| 0.665 | 0.016 | 0.442 | 0.07 | 18.823 | 0.099 | 0.078 |
| 1.302 | 0.024 | 0.489 | 0.02 | 15.349 | 0.194 | 0.15 |
| 0.217 | 0 | 0.061 | 0.045 | 13.594 | 0.131 | 0.106 |
| 0.049 | 0.004 | 0.032 | 0.036 | 14.949 | 0.194 | 0.16 |
| 0.077 | 0.016 | 0.02 | 0.024 | 17.129 | 0.165 | 0.119 |
| 0.268 | 0 | 6.01 | 0.006 | 13.803 | 0.135 | 0.111 |
| 0.115 | 0.12 | 0.042 | 0.04 | 15.03 | 0.142 | 0.118 |
| 0.083 | 0.009 | 0.022 | 0 | 18.171 | 0.264 | 0.219 |
| 0.068 | 0 | 0.012 | 0.005 | 17.956 | 0.211 | 0.175 |
| 0 | 0.01 | 0.079 | 0.026 | 14.442 | 0.123 | 0.103 |
| 0.126 | 0 | 0.03 | 0.032 | 10.121 | 0.168 | 0.141 |
| 0.071 | 0 | 0.042 | 0.037 | 14.21 | 0.23 | 0.196 |
| 0.251 | 0.015 | 0.049 | 0.008 | 14.179 | 0.153 | 0.124 |
| 0.614 | 0 | 0.373 | 0.007 | 14.169 | 0.344 | 0.277 |

| CLA trans-10 | Tagatose (m | Vitamin D2 ( | Vitamin D3 ( | Added Sugar | Total Grains | Whole Grain |
|---|---|---|---|---|---|---|
| 0.037 | 4.779 | 0.004 | 2.396 | 29.524 | 4.764 | 0.96 |
| 0.02 | 4.113 | 0 | 1.451 | 25.21 | 4.885 | 2.531 |
| 0.042 | 0.041 | 0.005 | 3.148 | 14.987 | 2.976 | 1.095 |
| 0.023 | 4.243 | 0.005 | 3.153 | 21.824 | 4.3 | 0 |
| 0.032 | 0.141 | 0.003 | 2.734 | 4.227 | 6.902 | 1.337 |
| 0.045 | 0.419 | 0 | 2.119 | 30.341 | 5.992 | 0 |
| 0.022 | 0.284 | 0 | 1.828 | 21.974 | 5.561 | 0 |
| 0.024 | 0.064 | 0 | 2.605 | 27.013 | 4.49 | 2.516 |
| 0.044 | 2.547 | 0.009 | 29.07 | 57.125 | 7.603 | 3.482 |
| 0.033 | 0.07 | 0 | 3.076 | 20.4 | 5.274 | 1.475 |
| 0.02 | 2.577 | 0 | 0.454 | 55.005 | 6.516 | 4.259 |
| 0.027 | 0.084 | 0.007 | 3.123 | 23.535 | 5.198 | 1.095 |
| 0.034 | 0.184 | 0.004 | 4.01 | 6.586 | 6.15 | 4.088 |
| 0.027 | 5.019 | 0.008 | 2.028 | 19.413 | 2.714 | 0.772 |
| 0.067 | 0.076 | 0.016 | 3.618 | 24.289 | 4.301 | 2.407 |

| Refined Grai | PUFA 18:3 n- | Solid Fats (g | Gluten (g) | Total Lignans | Secoisolarici | Matairesinol |
|---|---|---|---|---|---|---|
| 3.803 | 3.231 | 48.654 | 4.526 | 287.02 | 61.724 | 17.656 |
| 2.354 | 1.345 | 28.946 | 0.102 | 625.055 | 147.027 | 19.513 |
| 1.881 | 3.711 | 29.519 | 5.683 | 248.213 | 94.121 | 17.658 |
| 4.3 | 2.424 | 28.894 | 8.939 | 293.597 | 64.696 | 8.049 |
| 5.565 | 3.648 | 34.378 | 12.537 | 250.034 | 41.213 | 4.478 |
| 5.992 | 2.033 | 22.89 | 10.953 | 270.637 | 51.012 | 21.704 |
| 5.561 | 1.777 | 26.854 | 9.989 | 258.953 | 76.973 | 5.893 |
| 1.975 | 3.31 | 26.658 | 1.337 | 789.473 | 136.219 | 37.215 |
| 4.121 | 3.08 | 46.405 | 11.381 | 92.341 | 27.713 | 11.343 |
| 3.799 | 2.3 | 43.673 | 7.407 | 355.061 | 66.767 | 18.77 |
| 2.257 | 2.877 | 21.135 | 10.975 | 234.513 | 44.787 | 22.491 |
| 4.103 | 2.238 | 39.193 | 9.319 | 97.944 | 19.809 | 5.085 |
| 2.062 | 1.355 | 45.399 | 9.77 | 296.272 | 91.893 | 3.333 |
| 1.942 | 2.24 | 30.452 | 4.503 | 247.492 | 74.189 | 7.992 |
| 1.894 | 0.622 | 63.274 | 7.415 | 220.14 | 36.005 | 12.296 |

| Lariciresinol | Pinoresinol (ı | PUFA 18:2 n- | PUFA 18:3 n- | PUFA 20:4 n- | Omega-6 Fa | L-alanine |
|---|---|---|---|---|---|---|
| | | | | | | 22631260 |
| | | | | | | 28755920 |
| | | | | | | 17310800 |
| | | | | | | 26893510 |
| | | | | | | 13899240 |
| | | | | | | 22699350 |
| | | | | | | 28116130 |
| | | | | | | 22986500 |
| | | | | | | 21960900 |
| | | | | | | 17956830 |
| | | | | | | 19710610 |
| | | | | | | 22800500 |
| | | | | | | 16659230 |
| | | | | | | 13770880 |
| | | | | | | 22669640 |
| 134.502 | 73.091 | 10.993 | 0.019 | 0.258 | 11.27 | 18647380 |
| 379.864 | 79.326 | | | | | 15418360 |
| 95.487 | 41.221 | | | | | 15145050 |
| 168.309 | 54.286 | | | | | 19334350 |
| 157.86 | 48.691 | | | | | 20650440 |
| 129.607 | 69.976 | 18.702 | 0.012 | 0.158 | 18.873 | 15184070 |
| 154.73 | 22.794 | | | | | 21302720 |
| 370.999 | 245.741 | | | | | 19618230 |
| 39.863 | 13.676 | | | | | 23367800 |
| 244.559 | 26.644 | 15.741 | 0.022 | 0.27 | 16.033 | 12070830 |
| 97.227 | 70.063 | 25.821 | 0.048 | 0.125 | 25.995 | 9211699 |
| 50.787 | 22.51 | | | | | 13935690 |
| 161.434 | 39.77 | | | | | 13579010 |
| 126.622 | 40.115 | | | | | 14279520 |
| 151.047 | 20.951 | | | | | 20079150 |

| L-asparagine | L-aspartate | L-cysteine | L-glutamate | L-glutamine | glycine | L-histidine |
|---|---|---|---|---|---|---|
| 1090756 | 4556048 | 847254.8 | 1708571 | 26573970 | 2903077 | 2054609 |
| 1245005 | 7552644 | 1673297 | 3072769 | 26442890 | 2190493 | 3114031 |
| 1236245 | 5410274 | 970843.1 | 3192896 | 25745490 | 2681104 | 2763156 |
| 1096307 | 5943414 | 1481305 | 2737437 | 22372440 | 2868150 | 2452997 |
| 972282.3 | 3652699 | 1113193 | 2144563 | 23314650 | 3141750 | 2587948 |
| 1191077 | 939239.8 | 938302 | 2646708 | 28979040 | 4049766 | 2543664 |
| 1665126 | 3390708 | 771085.8 | 3485857 | 25492640 | 2692285 | 3032136 |
| 1236672 | 4039655 | 1213219 | 1805074 | 23405070 | 2930039 | 3018029 |
| 1027166 | 4574794 | 1166101 | 4290492 | 21638760 | 2901847 | 2760245 |
| 1031125 | 1041359 | 773795.5 | 2094749 | 22719070 | 3414611 | 2306615 |
| 1102969 | 5092162 | 1623315 | 1801875 | 28433080 | 3464063 | 2618216 |
| 1042514 | 4608970 | 1251587 | 4971272 | 23205430 | 2631619 | 2656731 |
| 1071279 | 5116458 | 1124464 | 3832078 | 24333790 | 3320162 | 2236018 |
| 999116.6 | 5402588 | 974101.2 | 1339799 | 19623800 | 2762562 | 2049612 |
| 1743352 | 4362644 | 1049491 | 2480766 | 28490410 | 3752939 | 2830973 |
| 1173193 | 1149092 | 1703860 | 7325680 | 26030350 | 1991439 | 3119436 |
| 1006006 | 3072547 | 1146870 | 5232238 | 24483830 | 2888847 | 2327045 |
| 1322068 | 969087.2 | 809073.7 | 3949473 | 28158010 | 3497964 | 2376205 |
| 1490507 | 1259819 | 1127719 | 4440966 | 28004900 | 3479671 | 3073623 |
| 1449410 | 840897.7 | 2113334 | 7507426 | 25651290 | 2872091 | 3008546 |
| 1440151 | 2958985 | 1144306 | 7797380 | 26583050 | 3581364 | 2331768 |
| 1463756 | 2740734 | 349500.8 | 37936210 | 9770084 | 3161277 | 2902507 |
| 985495.5 | 1115882 | 1144771 | 7123076 | 23919390 | 2403647 | 2715901 |
| 1145491 | 1931621 | 1811328 | 2160985 | 29062220 | 2769695 | 2407147 |
| 1071020 | 4652822 | 1476707 | 3905611 | 24227610 | 2483312 | 2132965 |
| 619016.4 | 1961123 | 795327.8 | 4415152 | 16735980 | 2284398 | 1621566 |
| 913934.6 | 970787.8 | 950315.7 | 3147806 | 19340270 | 3689266 | 1915791 |
| 1144611 | 2085332 | 964955 | 3852387 | 22666380 | 2552721 | 2477344 |
| 1004343 | 1320784 | 874369.8 | 1965897 | 22734040 | 3063102 | 2155166 |
| 1282976 | 1457972 | 597549.7 | 2468813 | 25548370 | 3301925 | 2619578 |

| L-leucine/iso | L-lysine | L-methionine | L-proline | L-serine | L-threonine | L-tryptophan |
|---|---|---|---|---|---|---|
| 45066640 | 4616760 | 7549364 | 55803920 | 1819290 | 6417908 | 39499360 |
| 78843390 | 9228431 | 15711290 | 116088500 | 2002434 | 12570750 | 48339010 |
| 58650760 | 7279232 | 10302380 | 67682230 | 1956129 | 8789476 | 51332720 |
| 66796260 | 8317108 | 13569150 | 95737750 | 1471156 | 9117863 | 54837240 |
| 37560400 | 4362728 | 9745749 | 89898140 | 1954944 | 8583357 | 48675280 |
| 48743730 | 6210228 | 12434600 | 91815110 | 2102536 | 7903886 | 51206640 |
| 51516610 | 7782342 | 12234640 | 72176220 | 1887347 | 10608570 | 38687460 |
| 66985720 | 8011292 | 11987450 | 138289600 | 1514143 | 11442980 | 70371960 |
| 64169870 | 6314110 | 11993720 | 123171700 | 1208570 | 6900532 | 73957140 |
| 55193960 | 5836172 | 10178060 | 67713220 | 2080388 | 8105796 | 35228970 |
| 49338840 | 4334982 | 8839483 | 59313830 | 1747576 | 6303224 | 47504140 |
| 54011140 | 5418986 | 9094804 | 65305630 | 1432146 | 6467552 | 45001100 |
| 44773040 | 4952432 | 9016785 | 83787220 | 2169533 | 6721676 | 46389160 |
| 48790090 | 4987545 | 9155688 | 54783540 | 1673318 | 4793321 | 30372200 |
| 42656160 | 4702116 | 9708397 | 97351700 | 1774098 | 9644540 | 45589040 |
| 69719140 | 9234771 | 11877070 | 70683050 | 2471689 | 8284928 | 55129320 |
| 42220240 | 6377920 | 8538912 | 50186590 | 2171072 | 6400558 | 35333860 |
| 55183520 | 6479146 | 9954980 | 66386720 | 2372660 | 9925693 | 37007680 |
| 42519890 | 5285160 | 8788839 | 149514100 | 2772770 | 13270470 | 53626570 |
| 47825990 | 7043336 | 9911269 | 106462900 | 2384897 | 10312170 | 72819670 |
| 52445600 | 5162236 | 9188528 | 98239870 | 2435857 | 10607050 | 41177030 |
| 46544330 | 6835906 | 11690060 | 108312200 | 2375421 | 6783772 | 40616170 |
| 80825030 | 6072760 | 10346220 | 77624900 | 1688912 | 6156310 | 50745870 |
| 67703870 | 7163406 | 13587580 | 108843400 | 1952854 | 9385849 | 77665720 |
| 42141450 | 4946150 | 8273878 | 64832010 | 1947345 | 7285616 | 49445440 |
| 36559240 | 4284554 | 5834256 | 38708450 | 1405605 | 4124330 | 27239070 |
| 51215880 | 5592826 | 8686873 | 68184200 | 2110992 | 5591157 | 47639220 |
| 42172480 | 5157338 | 8036914 | 91852900 | 1824407 | 6628132 | 42475160 |
| 51828180 | 5466966 | 9330142 | 64368800 | 1811327 | 6035650 | 36646760 |
| 44198950 | 5483612 | 9047055 | 97952900 | 1954570 | 7529190 | 38195720 |

| L-tyrosine | L-valine | L-cystine | ADP | AMP | Guanine | Cytosine |
|---|---|---|---|---|---|---|
| 15111550 | 79894410 | 670915.2 | 41538.69 | 34378.36 | 342172.9 | 40777.04 |
| 29955450 | 114126200 | 2266840 | 57758.5 | 47269.44 | 735415 | 56877.98 |
| 22615090 | 99375360 | 1048907 | 740.6837 | 529.1445 | 446647 | 52235.21 |
| 25533340 | 103929400 | 930832.9 | 0 | 1123.579 | 595568.9 | 64735.06 |
| 24547250 | 94134620 | 536879.4 | 3149.271 | 13761.41 | 431438 | 48494.29 |
| 24029920 | 111753200 | 680501.7 | 17981.59 | 1787.38 | 560997 | 54650.2 |
| 29300900 | 93497220 | 918120.2 | 55782.69 | 45223.41 | 567700.3 | 59554.12 |
| 31299510 | 115136700 | 1256099 | 26302.18 | 1909.665 | 645727.6 | 58957.57 |
| 28890550 | 133235900 | 1216822 | 38166.45 | 11435.21 | 567443.7 | 35188.84 |
| 18294730 | 111516800 | 1041140 | 28799.77 | 3031.628 | 320541.8 | 41358.16 |
| 20077490 | 83108020 | 1251203 | 17699.2 | 1588.81 | 392451.7 | 36022.46 |
| 19473470 | 96731780 | 598247.4 | 168760 | 86357.07 | 403082.5 | 31853.99 |
| 22373030 | 86998480 | 532107.4 | 58603.25 | 22875.14 | 426072.9 | 47725.59 |
| 20316150 | 81715050 | 862273.6 | 19402.07 | 8393.885 | 394840.7 | 44011.81 |
| 17091380 | 70447860 | 801783.8 | 56698.14 | 6370.412 | 393114.8 | 63251.87 |
| 29543470 | 121475700 | 1628869 | 181394.9 | 91315.18 | 449906 | 57924.25 |
| 19697090 | 76145480 | 1297654 | 99154.01 | 40345.57 | 368013 | 48938.19 |
| 20205990 | 117817300 | 1029332 | 152772.4 | 30782.87 | 449634.2 | 66952.35 |
| 23881990 | 83235870 | 980514.7 | 31133.23 | 28488.65 | 483626.5 | 37833.19 |
| 24921960 | 116734000 | 2211642 | 38776.16 | 36069.77 | 460855.2 | 61454.19 |
| 22421830 | 91175080 | 1036984 | 142759.2 | 37805.5 | 436035.3 | 66131.71 |
| 22973200 | 95869790 | 1024740 | 2352440 | 2194875 | 538324.4 | 110302.6 |
| 32420980 | 158478700 | 685291.2 | 155958.8 | 44674.12 | 497165.7 | 71610.04 |
| 39041160 | 143769000 | 2305428 | 28317.28 | 11206.12 | 621955.4 | 53826.37 |
| 17381460 | 76928870 | 1132841 | 31301.81 | 10195.55 | 317376.8 | 48605.79 |
| 15492100 | 58506690 | 1095645 | 66019.53 | 117186.8 | 264710 | 33739.98 |
| 19288070 | 95502940 | 1270156 | 149204.4 | 178178.9 | 411552.9 | 53427.53 |
| 20312880 | 74582150 | 737046 | 76013.32 | 58878.62 | 426433.9 | 63572.19 |
| 19943410 | 86692740 | 966565.2 | 91916.98 | 60559.9 | 406808 | 51271.09 |
| 23151330 | 84599980 | 796654.2 | 17451.09 | 42676.69 | 423094.2 | 42086.64 |

| Uracil | IDP | Inosine | Hypoxanthine | Xanthine | Allantoate | (S)(+)-Allant |
|---|---|---|---|---|---|---|
| 214614.2 | 1148357 | 22398.98 | 964731.2 | 192130.4 | 227143.3 | 47410.47 |
| 238959.7 | 1533806 | 15655 | 488195.4 | 144877.4 | 258080.6 | 56083.5 |
| 253741.2 | 1386755 | 20467 | 708671.2 | 114889.9 | 377921.3 | 80004 |
| 244167.5 | 1277096 | 12196.39 | 1009846 | 138072.2 | 269620.6 | 41337.13 |
| 200313 | 1208652 | 16942.51 | 680992.2 | 83144.34 | 304703 | 61200.18 |
| 264010.5 | 1543801 | 21908.69 | 1268404 | 109633.4 | 254642.3 | 64070.42 |
| 267547.5 | 884562.7 | 16468.84 | 971037.3 | 139613 | 441994.6 | 48711.86 |
| 282568.7 | 1410522 | 21979.7 | 863643 | 162661.4 | 231387 | 98761.55 |
| 236164.4 | 1603048 | 27498.34 | 1024201 | 112366.8 | 275141.3 | 71125.16 |
| 251183 | 1074852 | 30719.49 | 988760.5 | 154101.3 | 328067.5 | 118512.3 |
| 258485.3 | 1471326 | 13729.46 | 1320258 | 166234.7 | 311141.8 | 47740.6 |
| 268897 | 1026073 | 18232.58 | 842539.6 | 134043 | 240461.4 | 41757.48 |
| 250074 | 796477.7 | 20822.84 | 940114.3 | 158222.2 | 387146.5 | 65279.49 |
| 232018.8 | 1127956 | 22905.7 | 821112.7 | 121701.7 | 216125 | 50258.43 |
| 266822.1 | 2006964 | 6367.107 | 1210887 | 124068.3 | 197075.6 | 111084.1 |
| 263615.3 | 1585098 | 50610.03 | 1665039 | 190165.3 | 295410.5 | 54575.33 |
| 208995.6 | 1331382 | 25092.42 | 1021972 | 169875.3 | 313894.5 | 42920.88 |
| 266614.2 | 1571556 | 22244.94 | 1234192 | 239975.3 | 333193.4 | 56665.08 |
| 244193 | 1331169 | 25323.66 | 762107.1 | 151016.6 | 546551.3 | 83990.72 |
| 325994.9 | 2621035 | 47722.58 | 2305700 | 256324.4 | 681095.6 | 95839.06 |
| 242760.1 | 1061307 | 20365.11 | 1647915 | 140404.8 | 425136.7 | 60594.25 |
| 279407.2 | 1513081 | 20031.58 | 1943610 | 203831.1 | 798715.2 | 90259.59 |
| 241081.6 | 1424553 | 25228.74 | 1541186 | 194830.8 | 219298 | 66792.34 |
| 315878.9 | 1860967 | 19241.52 | 643875.8 | 347420 | 257406 | 120272.9 |
| 209476.2 | 1404237 | 15242.8 | 985142 | 149610.4 | 270122.9 | 64594.52 |
| 242445.6 | 1653587 | 28905.37 | 813136.3 | 109127.9 | 159932 | 57079 |
| 278046 | 1054995 | 22145.21 | 1435939 | 181360 | 191317.7 | 76087.32 |
| 249399.9 | 1688765 | 27575.71 | 1669585 | 169383.8 | 297560.7 | 105330.6 |
| 259361.2 | 1155226 | 16196.27 | 813214.4 | 148730.4 | 420696 | 36760.22 |
| 241593.2 | 1317122 | 22829.67 | 1682556 | 115304.4 | 525922.2 | 86035.82 |

| 5-Hydroxyiso | Urate | 3',5'-Cyclic IN | 5-6-Dihydrotl | 4-Pyridoxate | Pyridoxamin | Nicotinamide |
|---|---|---|---|---|---|---|
| 747110.2 | 48996720 | 57145610 | 3910684 | 18369.51 | 8543.745 | 161248.7 |
| 1022964 | 40592050 | 65616460 | 1582428 | 18214.01 | 16558.57 | 303966.2 |
| 660486.9 | 43635320 | 56956910 | 3302871 | 45790.75 | 71759.28 | 128399.4 |
| 841574.5 | 32412610 | 60402600 | 8355478 | 11555.93 | 35409.8 | 305227.3 |
| 648828.7 | 27720390 | 66118130 | 3922758 | 13156.11 | 37235.93 | 175944.8 |
| 651117.1 | 25677560 | 58799320 | 5993562 | 27745.19 | 90823.62 | 157596.3 |
| 539415.8 | 46585810 | 61157170 | 2894837 | 24092.79 | 5890.799 | 173329.5 |
| 888928.5 | 37833830 | 48265390 | 14211210 | 11783.79 | 11992.14 | 193846 |
| 637652.8 | 64122530 | 79789130 | 2044640 | 25684.79 | 14238.61 | 180166.5 |
| 715769.6 | 45208220 | 49419200 | 4718816 | 27863.78 | 21652.4 | 171238 |
| 740304 | 40492130 | 69780140 | 2770644 | 24310.01 | 26419.31 | 188578.5 |
| 545136.9 | 41821700 | 71862850 | 2445700 | 36331.45 | 17916.61 | 188035.3 |
| 688919.1 | 28304450 | 61817530 | 3524529 | 21873.98 | 18823.43 | 194298.9 |
| 534801.9 | 35845750 | 72403690 | 1390592 | 12629.02 | 6251.206 | 153275.6 |
| 744265.7 | 38098130 | 53507650 | 10121440 | 27962.64 | 51028.3 | 208979.7 |
| 736373.5 | 45011830 | 53638980 | 1913217 | 13559.9 | 40523.19 | 195375.2 |
| 605311.8 | 44242560 | 63901320 | 4318488 | 17439.39 | 4673.25 | 203428.6 |
| 631963.8 | 42274220 | 55235650 | 4465348 | 23357.41 | 86651.23 | 181059.4 |
| 591527.8 | 27562870 | 61069840 | 2897131 | 38242.25 | 30101.18 | 153443.5 |
| 534940.3 | 63960470 | 72981280 | 5826646 | 154202.8 | 172391.5 | 191034.7 |
| 629612.3 | 26265450 | 55739770 | 2632733 | 14655.61 | 25934.81 | 164648.5 |
| 361129.6 | 54364300 | 59006290 | 1804896 | 61811.17 | 45331.31 | 1985412 |
| 655760 | 36627260 | 53198640 | 3251660 | 37445.73 | 35421.27 | 268327.1 |
| 789196 | 56211150 | 53096080 | 14772440 | 31789.79 | 71689.91 | 272884.8 |
| 554377.2 | 53866460 | 65778720 | 5477235 | 10941.18 | 40862.63 | 226591.3 |
| 371315.7 | 48123310 | 90318550 | 1297595 | 8535.345 | 53611 | 174412.3 |
| 466125.5 | 48423500 | 83513080 | 3882435 | 25990.76 | 33398.14 | 263988.4 |
| 423877.6 | 43667390 | 55844130 | 12527360 | 18263.05 | 32061.41 | 167151.1 |
| 677511.9 | 22585820 | 49838160 | 1776502 | 29109.96 | 40156.81 | 221114.6 |
| 510556.5 | 30791430 | 65326030 | 5505994 | 73680.55 | 8994.652 | 440192.7 |

| Adenylosucci | Phosphate | Diphosphate | D-Glucose | D-Glyceralde | 1-3-Bisphosp | Pyruvate |
|---|---|---|---|---|---|---|
| 100981.2 | 11191240 | 65638.97 | 39193200 | 38232.14 | 55269.62 | 3359155 |
| 43173.43 | 12336220 | 143933.9 | 31425690 | 39726.19 | 46681.72 | 3767773 |
| 90085.49 | 13600420 | 147972.7 | 33065950 | 46084.08 | 20336.08 | 2378646 |
| 87168.41 | 13768230 | 95855.68 | 24518900 | 35628.3 | 19388.73 | 3786517 |
| 60220.91 | 11521480 | 157912.7 | 21735120 | 27400.26 | 24331.79 | 1873529 |
| 55190.75 | 14628070 | 159510 | 40753140 | 30998.18 | 8873.983 | 3203291 |
| 58217.56 | 14465270 | 81611.98 | 34656590 | 37103.29 | 15632.91 | 6033994 |
| 66770.43 | 13595740 | 163517.5 | 30039560 | 52964.46 | 15779.25 | 2461172 |
| 54053.21 | 16873640 | 179964.2 | 29928900 | 55300.76 | 36269.21 | 5139962 |
| 24912.26 | 13882130 | 110004.7 | 33246920 | 55518.84 | 224185.4 | 3485835 |
| 136949.5 | 14387610 | 150114.2 | 25385600 | 48468.06 | 16750.5 | 3242513 |
| 87936.27 | 10612910 | 105181.2 | 34412770 | 43156.62 | 6510.051 | 3651076 |
| 112755 | 10226490 | 78438.9 | 28710140 | 36462.49 | 15696.26 | 3112275 |
| 65589.46 | 12140710 | 135575.9 | 29169200 | 45549.12 | 13873.28 | 2445671 |
| 82064 | 18197870 | 251368.1 | 26111130 | 52118.75 | 20140.43 | 3244835 |
| 61499.61 | 16976650 | 204125.2 | 40886220 | 30244.79 | 120253.8 | 1793738 |
| 85070.59 | 15122260 | 124325.8 | 35956280 | 45229.53 | 246662.6 | 1257955 |
| 37577.91 | 17560040 | 197614.2 | 31810250 | 47487.51 | 660020.2 | 2830459 |
| 55404.11 | 18191370 | 196667.1 | 26474210 | 39544.84 | 16801.34 | 6955050 |
| 32426.65 | 28933490 | 320082 | 36383200 | 34190.49 | 24933.3 | 12507970 |
| 64684.25 | 13565090 | 138892.9 | 29594450 | 47184.61 | 146746.9 | 1578104 |
| 97974.77 | 15131450 | 280600.6 | 19295110 | 230227.4 | 13504050 | 4373174 |
| 18267.74 | 13265200 | 116429.2 | 26847590 | 21395.11 | 202486.9 | 1569755 |
| 24278.33 | 19072830 | 218396 | 32677900 | 57317.46 | 197801.6 | 1561266 |
| 78797.57 | 12733980 | 120865.8 | 29522910 | 38440.93 | 31634.77 | 3383385 |
| 66761.16 | 14612340 | 217700.3 | 32241500 | 50072.14 | 28519.94 | 2770015 |
| 31693.36 | 12066690 | 115429.9 | 26086940 | 31900.84 | 206628.6 | 3054437 |
| 97629.69 | 17832480 | 249654.1 | 29868160 | 47573.14 | 113257.8 | 2702723 |
| 38663.96 | 11332180 | 116989.9 | 23234190 | 49506.77 | 185536.7 | 3034597 |
| 47787.73 | 11656930 | 129007.5 | 31868390 | 61164.47 | 3611.342 | 5050591 |

| Lactate | Maltose | Mannitol | D-Ribose | D-Rhamnose | D-Arabitol | Citrate |
|---|---|---|---|---|---|---|
| 45187970 | 39580.65 | 428438.7 | 1094548 | 1241486 | 89701.79 | 14541150 |
| 75870460 | 10584.84 | 525928.1 | 919785.8 | 1465797 | 123969.9 | 12672350 |
| 30679360 | 28858.54 | 486449.3 | 921886.4 | 1646579 | 87519.29 | 7744702 |
| 53580130 | 27361.54 | 349867.2 | 700306.8 | 1637166 | 87773.32 | 11934440 |
| 22865220 | 26375.28 | 375211.8 | 652516.8 | 843074.8 | 73838.02 | 4688549 |
| 54895580 | 33814.63 | 344656.7 | 1142818 | 1693707 | 111561.5 | 14597000 |
| 92032680 | 53927.69 | 620504.5 | 950388 | 1833997 | 274072.4 | 9912044 |
| 30662130 | 49286.06 | 747543.5 | 865030 | 3288350 | 128454.4 | 12557180 |
| 98990780 | 25314.46 | 653698.6 | 863214.8 | 2823694 | 126358.8 | 5566580 |
| 46103500 | 41136.19 | 409995.9 | 903102.2 | 1516598 | 97265.09 | 12147010 |
| 61266520 | 33639.98 | 511191.7 | 735761.5 | 1842375 | 118159 | 10498220 |
| 62174830 | 96142.62 | 421447.1 | 962069.3 | 2559826 | 76244.19 | 7794870 |
| 52601820 | 29040.74 | 417490.4 | 840990.8 | 1546005 | 82945.11 | 8708797 |
| 41824780 | 55086.96 | 668780.8 | 837290.8 | 883478.1 | 70268.32 | 6336940 |
| 38665020 | 36146.43 | 414840.5 | 783196.6 | 2546788 | 123320.2 | 11636620 |
| 71186760 | 27705.72 | 536020.2 | 1170609 | 1413586 | 79965.55 | 13338340 |
| 38237550 | 42084.84 | 1203794 | 1023896 | 1001146 | 114617.5 | 10442320 |
| 68768060 | 29838.47 | 431419 | 924569.7 | 1202537 | 94428.77 | 12945190 |
| 90277300 | 113898.3 | 624105.6 | 706033 | 1306793 | 92021.98 | 9463216 |
| 154514500 | 46796.14 | 675136.2 | 987111.7 | 2316175 | 133381.8 | 22074680 |
| 34840730 | 30076.29 | 482873.9 | 866378.3 | 1900892 | 97434.85 | 6483370 |
| 161416700 | 533893.2 | 494581.9 | 508596.3 | 2244182 | 111792.8 | 11258020 |
| 49456480 | 38658.25 | 580695.8 | 757148.5 | 2661497 | 90319.16 | 6760342 |
| 53515540 | 38856.69 | 826095.3 | 793379.1 | 3956443 | 108981.4 | 15777330 |
| 50531070 | 25564.3 | 295392.2 | 779145.8 | 1560053 | 82736.51 | 9381389 |
| 51798240 | 18507.34 | 385734.5 | 903359.3 | 465891.3 | 67655.38 | 7742856 |
| 105292700 | 48394.04 | 345697.8 | 765184.2 | 1169490 | 82104.62 | 10779040 |
| 53208530 | 30645.6 | 448543 | 808421.2 | 2489134 | 102336.7 | 11613220 |
| 54052760 | 35971.94 | 497962.4 | 574483.6 | 1580702 | 75224.94 | 8820039 |
| 99628120 | 46861.11 | 634607 | 884893.8 | 1568925 | 87397.29 | 15891050 |

| 2-Oxoglutara | 2-Oxoglutara | Succinate | Fumarate | Malate | Oxaloacetate | 2-Hydroxyglu |
|---|---|---|---|---|---|---|
| 359903.8 | 58286.06 | 395858.5 | 82465.12 | 560338.5 | 142156.5 | 223369.2 |
| 302805.3 | 115671.2 | 400108.2 | 55979.54 | 485448.7 | 131894.2 | 111340.1 |
| 294494.4 | 91814.87 | 297338.3 | 54085.18 | 297050.5 | 98371.54 | 144812 |
| 542320.3 | 85330.42 | 411911 | 47884.69 | 401161.2 | 129521.5 | 192004.3 |
| 217851.4 | 69166.73 | 256633.4 | 51980.48 | 162280.9 | 93593.23 | 142655.6 |
| 458703.1 | 118175.2 | 387602.6 | 93435.69 | 466885.3 | 164623.8 | 206837.8 |
| 330716.1 | 113594.6 | 353446 | 77231.59 | 379644.5 | 118047.1 | 308386.6 |
| 226044.4 | 92923.53 | 266788.4 | 52903.08 | 268193.1 | 140498 | 182494 |
| 364917.5 | 93425.1 | 420633 | 39317.82 | 326861.3 | 146378.9 | 230023.2 |
| 338857.9 | 49880.47 | 268034.8 | 58155.81 | 487523.2 | 54614.61 | 179292.6 |
| 401104 | 91229.59 | 457277.1 | 70200.66 | 351285.5 | 119563.3 | 145417.7 |
| 308316 | 105366.5 | 380146.9 | 76352.71 | 391476.6 | 119191.2 | 168390.1 |
| 499826.8 | 78067.85 | 295281.9 | 67588.86 | 320963 | 165344.6 | 99534.44 |
| 293068.9 | 75610.2 | 227840.8 | 49203.57 | 302592 | 115863.2 | 221322.2 |
| 445100.3 | 84383.91 | 506971.5 | 61208.61 | 471420.2 | 142724.8 | 320534.2 |
| 329495 | 119008.5 | 391455.2 | 58346.72 | 349623 | 87042.02 | 143185 |
| 395777.1 | 81276.4 | 300021 | 58647.67 | 318397.8 | 123639.1 | 167844 |
| 512338.2 | 54190 | 251285.2 | 92290.71 | 489744.2 | 135485.5 | 376697.3 |
| 440555.3 | 105235.1 | 325587.6 | 72610.32 | 351534 | 116307.7 | 198684.5 |
| 828307 | 211930.4 | 467188.4 | 73522.12 | 748924.3 | 182009.1 | 176282.9 |
| 318714.5 | 101650.8 | 231309.5 | 45176.52 | 180093.9 | 113322.8 | 83278.11 |
| 1498422 | 131158.6 | 911867.1 | 154655 | 1308272 | 139894.5 | 347848.4 |
| 241819.8 | 95710.65 | 250253.4 | 46714.46 | 226267 | 137843 | 170016.5 |
| 263610.3 | 95228.85 | 222674 | 68108.91 | 310018.5 | 170013.9 | 96367.72 |
| 547326.3 | 94702.21 | 291222.5 | 32713.77 | 382303 | 147504 | 178569.7 |
| 482909.3 | 74027.03 | 312801.6 | 56264.45 | 210559.5 | 113666.6 | 195656.8 |
| 446002.8 | 81404.16 | 289213.2 | 56597.56 | 453034.1 | 135869.4 | 188440.8 |
| 411171.9 | 77656.12 | 275866.5 | 87377.31 | 378547.7 | 131956.2 | 190766.9 |
| 264806.4 | 91854.38 | 314601.9 | 33716.46 | 337782.1 | 126529.9 | 181400 |
| 404584.1 | 132843.2 | 510134.3 | 99282.81 | 507035.8 | 119533.4 | 162183.5 |

| D-Glucono-1 | Glutathione | 5-Oxoproline | S-Glutathion | Cys-Gly | Ascorbate | gamma-Glut |
|---|---|---|---|---|---|---|
| 65210 | 29612.14 | 16602750 | 25138.06 | 771179.8 | 336896.4 | 98556.88 |
| 145055.3 | 8261.397 | 12550540 | 22744.02 | 756659.2 | 299972.7 | 80787.93 |
| 130908.8 | 10457.19 | 15854680 | 31896.67 | 742319.8 | 252223.4 | 150989 |
| 129942.5 | 47076.86 | 11165110 | 0 | 1009809 | 202060.5 | 82170.52 |
| 54624.88 | 19792.15 | 14612580 | 8726.24 | 613628.1 | 173039.7 | 146435.5 |
| 137992.2 | 49066.14 | 17277380 | 8454.217 | 679533.9 | 350231.8 | 116078.3 |
| 74400.48 | 16840.51 | 16300560 | 31630.31 | 826187.5 | 265538.5 | 183349.7 |
| 113116.4 | 0 | 12118760 | 15574.99 | 866276.8 | 326233.8 | 91990.01 |
| 122883.8 | 11016.96 | 14923080 | 8858.279 | 776833.2 | 278066.9 | 112921.1 |
| 148497.7 | 37172.03 | 14034080 | 30229.01 | 491213 | 285691.4 | 120914.7 |
| 97853.46 | 43184.19 | 17091790 | 36653.54 | 979494 | 236769.2 | 121328.1 |
| 60563.91 | 49840.04 | 14231480 | 12975.82 | 721828 | 263795.4 | 94230.45 |
| 65844.15 | 31322.43 | 16702610 | 0 | 1048546 | 251000.9 | 151373.3 |
| 86183.93 | 19617.67 | 12211930 | 19645.08 | 547721.1 | 267912.7 | 75242.43 |
| 40268.5 | 51826.71 | 17216510 | 41523.84 | 950460.8 | 240685.7 | 92557.01 |
| 114369.8 | 0 | 16127210 | 0 | 752215 | 361629.7 | 99951.28 |
| 116897 | 7410.144 | 15345000 | 21476.54 | 566329.6 | 319505.7 | 140876.4 |
| 124492.8 | 57606.71 | 17927100 | 19359.73 | 704893.8 | 272417.1 | 181691.1 |
| 92928.45 | 29052.02 | 16718450 | 32769.87 | 816492.8 | 212239.4 | 270093 |
| 117747.2 | 55877.21 | 17506710 | 30520.9 | 1585596 | 349511.2 | 325879.9 |
| 52265.94 | 9845.249 | 16317310 | 2917.595 | 834178.5 | 244039.1 | 156448.2 |
| 120790 | 339533.3 | 14170720 | 202080.3 | 1285490 | 151234.5 | 322407.3 |
| 101732.6 | 37173.29 | 11983420 | 7663.489 | 691723.7 | 216684.5 | 121289.9 |
| 51298.83 | 28317.53 | 14320100 | 23960.75 | 1178046 | 241366 | 123231.8 |
| 90985.6 | 4073.777 | 15514540 | 18830.88 | 687872.2 | 239981.5 | 113484.6 |
| 32997.52 | 7488.711 | 10922100 | 0 | 335079.2 | 325406.9 | 67444.99 |
| 61339.72 | 42713.57 | 11542930 | 20854.86 | 772280 | 222887.4 | 72571.72 |
| 41779.97 | 37205.04 | 13524600 | 18757.66 | 610771.6 | 244275.1 | 90168.5 |
| 101024.4 | 57667.41 | 13606810 | 74035.47 | 681761.8 | 190317.6 | 190483.2 |
| 85292.78 | 27031.76 | 15998430 | 45687.21 | 685230.4 | 272823 | 229570 |

| gamma-L-Gl | gamma-Glut | (5-L-Glutamy | L-Homocyste | Dimethylglyc | Ornithine | L-Citrulline |
|---|---|---|---|---|---|---|
| 548590.1 | 45188.19 | 1089853 | 692258.2 | 2692386 | 945548.3 | 2187974 |
| 608627 | 36103.23 | 835099.3 | 351470.2 | 5388106 | 1450562 | 2324528 |
| 597685.4 | 46232.15 | 955746.4 | 280510.2 | 5953616 | 1263983 | 3208151 |
| 767489.8 | 36930.58 | 881811.4 | 384265.5 | 2881490 | 1550056 | 1921357 |
| 464256.9 | 43690.78 | 889017.3 | 264884.9 | 3036179 | 1266754 | 2575138 |
| 502121.2 | 17700.9 | 1139336 | 936532.8 | 3401949 | 1107905 | 3453267 |
| 606725.2 | 28260.03 | 970132.1 | 420367.8 | 4451154 | 1616944 | 2904828 |
| 637882.4 | 54596.75 | 954959.2 | 742034.2 | 2377426 | 1318999 | 2278449 |
| 687102.1 | 73039.45 | 826033.6 | 229952.1 | 3094494 | 1235692 | 3255647 |
| 266040.8 | 18863.48 | 637132.3 | 286662 | 4551718 | 1150200 | 2477678 |
| 548536.4 | 40678.9 | 1178872 | 294166.2 | 2756148 | 1092902 | 2660778 |
| 468251.4 | 41515.18 | 812275.5 | 323269.2 | 3602947 | 1038572 | 2020666 |
| 493666.2 | 20983.64 | 826977.2 | 452107.3 | 2378049 | 1525889 | 2959232 |
| 485800.1 | 34196.29 | 628774.5 | 238806.8 | 2402440 | 922340.8 | 2965015 |
| 594824.7 | 36974.44 | 1206290 | 477220.9 | 2411271 | 920889.7 | 3239428 |
| 273543 | 13719.32 | 689635.8 | 410630.6 | 7482574 | 1469459 | 2429423 |
| 300053.4 | 29671.97 | 693343.7 | 612886.2 | 3878591 | 1335628 | 2595853 |
| 200266.4 | 36949.11 | 591201.8 | 518659.2 | 3654817 | 1515786 | 2683717 |
| 394667.4 | 33410.54 | 1249062 | 322332.6 | 2681573 | 1969601 | 2768493 |
| 488622.6 | 63346.21 | 1266530 | 367819 | 7078172 | 3045171 | 3728008 |
| 304746.3 | 13525.62 | 744519.9 | 211042.6 | 2272901 | 1533852 | 3790138 |
| 175618.5 | 114012.1 | 94826.56 | 253570.8 | 4500692 | 3186489 | 3237461 |
| 289224.7 | 34950.12 | 452876 | 581263.8 | 5729478 | 1240091 | 2206866 |
| 488582.7 | 37660.25 | 994036.8 | 370826.8 | 4895388 | 1337762 | 2464980 |
| 407052.5 | 45758.25 | 658585.6 | 312309.1 | 3440023 | 1132465 | 2492231 |
| 129510.7 | 16258 | 406369.5 | 191473.5 | 2259182 | 743124 | 2743524 |
| 196946 | 45668.68 | 543104.9 | 231097.7 | 3892142 | 1228573 | 2040288 |
| 316352 | 43025.73 | 766475.6 | 461055.3 | 3877043 | 907632.7 | 3303482 |
| 246679.2 | 29642.68 | 842893.4 | 251202.7 | 2816252 | 1685844 | 2346319 |
| 666226.9 | 45396.72 | 1396462 | 623054.1 | 3045210 | 1753012 | 2984904 |

| Cadaverine | Spermidine | N-Acetylneu | alpha-D-Gluc | D-Glucosami | Phosphocrea | Creatine |
|---|---|---|---|---|---|---|
| 5847.656 | 35184.72 | 54634.78 | 24427.8 | 257908.1 | 191613.2 | 13281940 |
| 16909.26 | 0 | 18874.01 | 13620.14 | 308610.5 | 79490.15 | 51442900 |
| 5085.46 | 17069.24 | 39824.03 | 33395.7 | 253785.8 | 153744.6 | 32392750 |
| 15996.45 | 16032.48 | 22399.24 | 21095.67 | 288318.2 | 216424.5 | 15122440 |
| 15122.06 | 25347.16 | 22572.71 | 24334.52 | 268077.8 | 124654.9 | 13948960 |
| 88932.04 | 17296.72 | 37829.26 | 30976.03 | 187827.5 | 158614.2 | 18006970 |
| 9562.999 | 10832.79 | 28591.28 | 15395.71 | 182258.3 | 96467.63 | 31612030 |
| 17084.31 | 11259.22 | 54669.02 | 15810.97 | 238256.7 | 157347 | 6591188 |
| 10793.96 | 44179.36 | 48498.38 | 35006.97 | 255047.7 | 88090.49 | 44531030 |
| 189481.3 | 27632.78 | 30645.51 | 16387.5 | 190835.8 | 107208.8 | 48994560 |
| 5377.016 | 7769.055 | 15940.87 | 21240.1 | 290236.4 | 148404.1 | 6790426 |
| 7013.331 | 8955.611 | 45443.19 | 28071.55 | 255240.1 | 122339.2 | 18961500 |
| 17951.02 | 12283.72 | 37057.25 | 25154.96 | 275439.7 | 96666.45 | 10842200 |
| 3549.87 | 42420.76 | 14394.4 | 14296.3 | 298270.5 | 110263.2 | 23270130 |
| 8356.08 | 12193.21 | 43645.43 | 10511.87 | 184609.8 | 146377.7 | 3872087 |
| 30834.81 | 9285.023 | 50702.55 | 19070.94 | 406390.6 | 80537.4 | 66676990 |
| 27229.44 | 19475.79 | 30353.24 | 28269.34 | 285644 | 53120.61 | 27478630 |
| 7376.916 | 13267.77 | 42662.89 | 32994.37 | 332246.3 | 141172.7 | 27191830 |
| 7836.539 | 18856.8 | 31773.95 | 5928.028 | 201598.2 | 99417.68 | 20359110 |
| 52665.95 | 2364.829 | 82439.45 | 35168.08 | 195963.6 | 80463.38 | 52564420 |
| 5861.583 | 7204.977 | 27305.53 | 15938.72 | 187748.6 | 63480.89 | 23060390 |
| 12107.43 | 1231774 | 138547.3 | 93224.72 | 227210.5 | 214965.7 | 57376760 |
| 9669.247 | 1488.823 | 34407.59 | 17731.81 | 317025.9 | 75214.79 | 32692840 |
| 6818.156 | 9212.605 | 54316.81 | 43340.84 | 209375.3 | 201100.6 | 18016910 |
| 13394.03 | 20077.2 | 44548.48 | 29533.98 | 309594 | 108533.5 | 28476130 |
| 3879.515 | 22166.45 | 36138.26 | 8985.164 | 297778.9 | 50307.95 | 30421620 |
| 39578.2 | 71897.59 | 48819.83 | 29551.97 | 235411.8 | 72640 | 22117910 |
| 5157.824 | 5760.714 | 60947.17 | 5860.203 | 213712.1 | 79449.34 | 13388990 |
| 6370.49 | 52582.48 | 33781.52 | 8936.796 | 197538.2 | 183739.6 | 13624420 |
| 147405.1 | 12463.07 | 52947.88 | 14159.97 | 268411.8 | 131712.8 | 30686510 |

| Creatinine | 4-Acetamido | N-Acetylorni | Guanidinoac | trans-4-Hydr | Pantothenate | Taurine |
|---|---|---|---|---|---|---|
| 49010290 | 823219.5 | 962554 | 788174.5 | 1151065 | 37688.36 | 4441792 |
| 39612850 | 173007.5 | 808966.3 | 618093.1 | 1281436 | 72328.98 | 3996787 |
| 45152980 | 350194.8 | 431849.8 | 603439.9 | 1056955 | 93939.87 | 5129546 |
| 50091460 | 167144.4 | 663308.8 | 767671.7 | 1709064 | 31234.12 | 6335964 |
| 33068470 | 1381694 | 522682.7 | 796625 | 1510206 | 25448.2 | 4032342 |
| 49112380 | 246414.6 | 780828.8 | 1080670 | 1044847 | 138517.7 | 6022102 |
| 40849160 | 447583.3 | 581549.3 | 688022 | 1497218 | 61961.62 | 5343974 |
| 50988080 | 244320.2 | 888869.5 | 541326.2 | 1924754 | 62030.72 | 5336624 |
| 32148470 | 1911793 | 644620.6 | 566252.9 | 1206826 | 39445.77 | 4812118 |
| 50361870 | 427590.2 | 702490.8 | 544633.8 | 1636610 | 41104.86 | 5285146 |
| 47088490 | 118853.5 | 566789.4 | 795362.4 | 951989.2 | 69734.45 | 4051385 |
| 36684760 | 345339.8 | 559671.8 | 775962.4 | 1202972 | 95059.16 | 5764308 |
| 43019880 | 145933.2 | 648922.8 | 825975.1 | 1291488 | 59128.73 | 5900664 |
| 39409520 | 142517.5 | 539006.9 | 648484.6 | 1873019 | 37177.19 | 3408543 |
| 58327370 | 290433.2 | 1485871 | 793443.4 | 1330948 | 38672.68 | 5608048 |
| 36565810 | 96105.4 | 398218.9 | 702156.2 | 1399080 | 90855.27 | 6869540 |
| 44542850 | 717527.3 | 274349.3 | 844259.7 | 893355.3 | 61416.78 | 5534039 |
| 49357790 | 712449 | 337140.2 | 1066951 | 894612.3 | 52430.49 | 7099684 |
| 31419180 | 1537262 | 406500.8 | 959694.6 | 1069749 | 28410.07 | 4233306 |
| 73997600 | 1352231 | 190402.8 | 1041085 | 1360690 | 218353.5 | 14433140 |
| 39066540 | 294711.5 | 395136.5 | 913011.1 | 2019491 | 67237.94 | 6097378 |
| 29719200 | 1073568 | 393105.7 | 587962.7 | 1356476 | 73858.54 | 19588460 |
| 41662160 | 230554.8 | 503189.8 | 692478.9 | 1260392 | 105778.7 | 4538688 |
| 69930550 | 158772.6 | 485740.2 | 866888 | 2865913 | 65176.03 | 6204258 |
| 48433320 | 431347.5 | 656134.2 | 861809 | 1783658 | 52686.45 | 6546762 |
| 27931350 | 487677.2 | 329096.4 | 456077.7 | 795401.8 | 58301.14 | 3886527 |
| 44333380 | 78662.62 | 589202.1 | 562766.8 | 1380009 | 53288.44 | 7470120 |
| 50175860 | 187130 | 779252.8 | 685253.2 | 1013872 | 53436.56 | 5852258 |
| 34972800 | 57497.72 | 425540.2 | 582715.4 | 2688743 | 40667.96 | 4184130 |
| 39357920 | 1287289 | 754246.2 | 722155.8 | 1359669 | 114445 | 6501238 |

| Hypotaurine | 3-Sulfino-L-a | Thiocysteine | 3-Sulfocatecl | (R)-S-Lactoyl | L-Methionine | 5-Hydroxyind |
|---|---|---|---|---|---|---|
| 4236.558 | 36617.3 | 11919.83 | 1192396 | 214201.8 | 31814.41 | 3794.827 |
| 20512.81 | 85473.65 | 31265.18 | 84632.2 | 190218.2 | 31950.06 | 4246.693 |
| 11667.17 | 51742.11 | 17810.47 | 546962.8 | 236327.7 | 28508.88 | 9221 |
| 6468.369 | 76475.84 | 15766 | 1213557 | 128925.8 | 25606.05 | 9778.245 |
| 5302.96 | 52431.68 | 10163.21 | 1684237 | 130810.4 | 17135.92 | 6815.99 |
| 11389.1 | 48414.86 | 13596.33 | 320486.2 | 207772 | 33461.32 | 1885.599 |
| 17120.09 | 42305.4 | 11952.86 | 737923.2 | 176152.8 | 32828.77 | 1055.733 |
| 8793.878 | 58405.29 | 23788.13 | 769852.3 | 88568.79 | 28196.15 | 9761.375 |
| 16990.18 | 52383.48 | 21122.7 | 1615024 | 189485.2 | 33623.41 | 5722.461 |
| 16869.43 | 42474.18 | 18673.45 | 351399.1 | 187573.8 | 28178.21 | 9728.796 |
| 12889.32 | 103625.7 | 24481.63 | 2769451 | 192590.5 | 23485.55 | 23283.38 |
| 14011.56 | 49401.14 | 10330.44 | 963010.7 | 197295 | 19970.51 | 2070.679 |
| 10350.64 | 84239.8 | 5732.406 | 2431216 | 267860.6 | 20895.44 | 9567.884 |
| 4737.42 | 25427.97 | 5901.311 | 400650.3 | 190906.6 | 26285.69 | 5528.086 |
| 4837.458 | 44691.97 | 13557.22 | 781271.8 | 285934.5 | 24868.09 | 6777.691 |
| 25825.74 | 88249.82 | 22199.85 | 108903.7 | 323827.2 | 21268.38 | 10302.4 |
| 13594.87 | 59949.37 | 25478.91 | 1120976 | 247373.1 | 16936.93 | 4692.822 |
| 4414.232 | 41908.81 | 16084.05 | 312905.5 | 314256.9 | 24348.02 | 11659.79 |
| 15892.68 | 49377.93 | 17905.42 | 981194.9 | 169835.4 | 27232.45 | 6941.328 |
| 32115.43 | 91621.47 | 29581.5 | 888732 | 144476 | 31042.19 | 22986.11 |
| 14715.94 | 81477.32 | 14425.26 | 2294835 | 296253.2 | 25909.52 | 6357.051 |
| 98264.44 | 33320.27 | 16215.21 | 584483.2 | 53429.23 | 26062.86 | 63292.93 |
| 11913.01 | 47056.8 | 10315.26 | 434060.3 | 244808.9 | 23329.28 | 3209.698 |
| 14471.83 | 83854.45 | 30742.38 | 569753.7 | 162531.2 | 30261.15 | 15381.84 |
| 16102.5 | 70114.3 | 16932.51 | 791992.2 | 180037.8 | 28079.71 | 6891.314 |
| 18996.03 | 54037.38 | 17050.79 | 442255 | 162333.9 | 20734.91 | 7115.833 |
| 27777.22 | 46755.19 | 25781.03 | 623637.1 | 96278.06 | 32934.52 | 9040.363 |
| 22131.43 | 36111.14 | 14039.12 | 487296.2 | 243460.6 | 32638.69 | 7135.816 |
| 6099.475 | 51967.77 | 16416.65 | 184971.4 | 192509 | 18935.97 | 6708.993 |
| 16161.12 | 36987.21 | 6986.041 | 854285.5 | 155410.4 | 30325.83 | 6609.065 |

| Indole | quinolinic aci | g-Oxalo-crot | L-Adrenaline | Serotonin | Ectoine | 3D-(3-5/4)-T |
|---|---|---|---|---|---|---|
| 53726.64 | 413808.6 | 494356.3 | 3837760 | 107722.8 | 203544.7 | 348046.4 |
| 47611.07 | 371351.1 | 577143.4 | 6031290 | 93913.66 | 153902.7 | 310988.4 |
| 67333.88 | 372487.3 | 186046.3 | 4284296 | 139753.9 | 195152 | 286315.2 |
| 58242.81 | 305475 | 583333.3 | 4432928 | 79427.13 | 130914 | 279468 |
| 72417.66 | 227840.7 | 311418.5 | 3295801 | 90199.52 | 128597.2 | 205192.6 |
| 62811.55 | 220060.1 | 359786.6 | 4841270 | 183060.1 | 80068.71 | 377935.6 |
| 68534.2 | 400125.5 | 372334.8 | 4657367 | 124978.5 | 227148.1 | 364530 |
| 77151.4 | 360601.5 | 91620.44 | 3898303 | 132735 | 156967.6 | 316511.9 |
| 84109.77 | 444667.8 | 250921.4 | 5162880 | 131969.4 | 400873.9 | 297178.5 |
| 51164.89 | 420847.9 | 389713.6 | 3631448 | 115060 | 86179.35 | 333126.2 |
| 65921.84 | 295937 | 267056.4 | 3009208 | 83962.45 | 126843.6 | 256655.4 |
| 63681.14 | 338019.3 | 128181.9 | 4992338 | 80048.49 | 208237 | 319849.2 |
| 61892.73 | 283564.9 | 522691 | 2812814 | 120574.1 | 110496 | 292960.8 |
| 54815.2 | 328596.8 | 312111.8 | 4567394 | 122102.2 | 139216.1 | 258048.5 |
| 42126.49 | 354078 | 174910.2 | 3500364 | 126903.2 | 143385.5 | 275922.3 |
| 49345 | 332473.8 | 1793598 | 6126540 | 92209.55 | 160292.8 | 442325.4 |
| 52813.61 | 370779.9 | 1228869 | 3933585 | 118331.1 | 91313.94 | 391388.2 |
| 46605.14 | 400547.2 | 1421401 | 3763106 | 80261.35 | 190511.6 | 279520.7 |
| 66036.18 | 248611.6 | 513507.9 | 3695668 | 112771.1 | 116926.6 | 290520.1 |
| 78830.04 | 478920.2 | 812153.4 | 5406410 | 188663.1 | 118340.8 | 369366.1 |
| 51052.36 | 206426.5 | 1739758 | 4057379 | 101315.1 | 86316.27 | 288464.5 |
| 61667.76 | 440770.8 | 1268330 | 3098729 | 109849.7 | 130486.8 | 193330.4 |
| 66341.22 | 306813.3 | 1539789 | 5997498 | 71577.01 | 212507.4 | 275533.2 |
| 86343.61 | 514195.8 | 556246.6 | 5520470 | 109297.2 | 267274.8 | 239823.9 |
| 52029.03 | 460390.6 | 481830.2 | 4232958 | 87295.19 | 127219.2 | 269099.2 |
| 42479.29 | 379734.7 | 420868.3 | 3940308 | 135109.5 | 94952.62 | 373484.3 |
| 70553.98 | 393350.2 | 1595967 | 4089123 | 98841.16 | 105109.4 | 223108.1 |
| 51799.83 | 385867.9 | 1173284 | 3415890 | 150840.8 | 75286.85 | 301397 |
| 54696.75 | 179727 | 898716.8 | 2872757 | 94044.63 | 57221.46 | 184288.5 |
| 59648.71 | 274468.5 | 32281.78 | 4188714 | 114263.2 | 108844.4 | 262301.6 |

| Ethanolamin | N-Methyleth | Acetylcholine | Choline | L-Carnitine | acyl-C2 (acet | acyl-C3 (prop |
|---|---|---|---|---|---|---|
| 43362.32 | 3803768 | 1660734 | 10452040 | 36774800 | 26489340 | 563576.6 |
| 40575.64 | 5399332 | 1891420 | 22314680 | 73901580 | 40033410 | 1909076 |
| 100082.7 | 4598432 | 1546267 | 11751230 | 47078930 | 25841910 | 1006519 |
| 54351.89 | 4392572 | 1740302 | 9515908 | 52936750 | 24452900 | 1386150 |
| 40710.04 | 4478292 | 1639733 | 12019860 | 34485740 | 24732210 | 788962.7 |
| 40468.43 | 3881182 | 1391043 | 13243610 | 49623660 | 34136590 | 855665.2 |
| 51450.3 | 2135297 | 1297280 | 13148170 | 42104880 | 13488740 | 535839.2 |
| 126638.2 | 4936208 | 2103683 | 11464420 | 37561030 | 31433880 | 1065887 |
| 43318.55 | 4471560 | 1142210 | 11347330 | 55772030 | 17837870 | 1153262 |
| 104934.5 | 3639336 | 1602773 | 13584410 | 46856360 | 37004780 | 1384590 |
| 34006.94 | 4491302 | 1303237 | 14568710 | 30303650 | 20133270 | 623809.9 |
| 95570.45 | 2882672 | 1029943 | 10908240 | 42380730 | 16782040 | 723004.3 |
| 58017.73 | 3946207 | 1142529 | 8743771 | 27485140 | 19857400 | 484170.5 |
| 41617 | 4017995 | 1335511 | 7407800 | 37811880 | 20230970 | 816427.8 |
| 95207.74 | 4134805 | 1229817 | 12741310 | 34547120 | 20059630 | 741085.2 |
| 99597.49 | 5213814 | 1624572 | 23675680 | 73906860 | 41381650 | 2478791 |
| 39489.92 | 3067557 | 1093564 | 10425220 | 33847110 | 21437640 | 537290.8 |
| 121581 | 3483586 | 1387224 | 13983900 | 35964450 | 44985170 | 733219.2 |
| 60872.35 | 3339075 | 2015895 | 15854780 | 38636240 | 19433390 | 734679.1 |
| 121375.4 | 4295358 | 1930939 | 20223470 | 62660440 | 51572240 | 1579513 |
| 85813.55 | 3542719 | 1530162 | 11446310 | 41083580 | 24696930 | 659592.1 |
| 932815.6 | 5056238 | 1943614 | 15853980 | 50754330 | 37546830 | 1885994 |
| 24077.58 | 3521916 | 1409755 | 12496390 | 50511140 | 38413920 | 1412537 |
| 63486.47 | 3587969 | 2696076 | 16466460 | 63255950 | 59383000 | 1342242 |
| 98471.4 | 3915900 | 1474465 | 16625120 | 48027800 | 43760500 | 852316.8 |
| 59029.8 | 3012406 | 667601.4 | 8366634 | 30505030 | 15272860 | 517101 |
| 50860.07 | 3325585 | 1528294 | 20902080 | 39820590 | 32431560 | 1088708 |
| 48088.2 | 2824855 | 1216357 | 12401590 | 33950390 | 26620070 | 711151.1 |
| 46080.07 | 3754990 | 1106250 | 9018321 | 28837260 | 15728030 | 580860.6 |
| 80638.31 | 3472371 | 1370459 | 11514760 | 52126410 | 21369930 | 849144.2 |

| acyl-C4 (buta | acyl-C5 (isova | acyl-C5:1 (Tig | acyl-C6 (hexa | acyl-C8 (L-oc | acyl-C8:1 (oc | acyl-C10:1 (C |
|---|---|---|---|---|---|---|
| 55355.28 | 631119.1 | 81884.43 | 175238.7 | 2691813 | 956709.3 | 1450205 |
| 107748.3 | 1148198 | 193975.5 | 384130 | 1957325 | 2195206 | 1488809 |
| 68531.24 | 695712.6 | 104350.7 | 159126.7 | 719577.4 | 2120223 | 1219923 |
| 97734.28 | 539775.9 | 71443.18 | 142086.8 | 938127.2 | 1304282 | 615642.5 |
| 49143.43 | 533670.5 | 78224.47 | 178787.3 | 1049164 | 615807.8 | 952071.3 |
| 34530.83 | 510388.5 | 63576.68 | 253468.3 | 1762156 | 1288989 | 1568106 |
| 45142.03 | 823824.8 | 98132.12 | 108182.7 | 1089548 | 557546 | 876463 |
| 131898.9 | 1140858 | 136833.8 | 136502.8 | 721651.4 | 1675129 | 764125.5 |
| 38308.49 | 747560.8 | 115182.5 | 63331.61 | 226421 | 694137.6 | 264516.1 |
| 58544.54 | 1132398 | 147759.2 | 349716.3 | 2848717 | 1153780 | 1643560 |
| 20503.39 | 330562.3 | 42507.15 | 196037.3 | 2163030 | 951129.8 | 1628429 |
| 66411.91 | 424198.4 | 120264.9 | 118929.5 | 891677.7 | 545385.9 | 885889.4 |
| 24335.37 | 400122.6 | 36679.47 | 133149 | 1193826 | 787334.4 | 879698 |
| 50087.64 | 475084.8 | 53907.31 | 121173 | 668549.5 | 1771459 | 784698.1 |
| 52959.78 | 512806 | 101692.1 | 240706 | 2884506 | 1635211 | 3032760 |
| 88085.88 | 1356755 | 199963.5 | 329415.3 | 1297019 | 3547069 | 1428594 |
| 32952.54 | 517128 | 57829.94 | 123418.3 | 1154563 | 760644.6 | 650397.9 |
| 89215.53 | 956010.1 | 67922.7 | 241195 | 2267073 | 1107161 | 1040004 |
| 48358.14 | 1513726 | 52210.81 | 191105.2 | 938017.3 | 1000575 | 934793.3 |
| 46287.06 | 1590460 | 142245.5 | 304770.6 | 1460308 | 4172136 | 1592281 |
| 60482.75 | 609098.2 | 48119.39 | 146038.3 | 1061271 | 620952.6 | 413019.6 |
| 33884.55 | 478492.5 | 62690.46 | 155443.1 | 676654.1 | 3056451 | 700216.3 |
| 134538.2 | 764673.2 | 165074.5 | 200742.4 | 1386693 | 2974791 | 1336878 |
| 108400.1 | 1231074 | 217043.4 | 262834.1 | 1540837 | 2123679 | 1258099 |
| 73414.26 | 725562.2 | 133565.7 | 260578.2 | 2497861 | 2428434 | 1142125 |
| 56952.18 | 508900.1 | 76811.53 | 99793.66 | 436191.2 | 1436922 | 414100.5 |
| 45133.36 | 661267.1 | 63616.36 | 125326.7 | 1061992 | 742622.2 | 682800.9 |
| 39757.26 | 601667.9 | 130225.2 | 157893.4 | 1143545 | 1156713 | 1029324 |
| 15423.66 | 309314.3 | 46072.39 | 142930.5 | 1133192 | 563758 | 782229.3 |
| 56358.2 | 547115.9 | 67345.91 | 134978.1 | 1161458 | 725680.3 | 877741.2 |

| Nonanoic aci | Decanoic aci | Dodecanoic a | Tetradecanoi | Octadecanoi | Tetradecenoi | Hexadecenoi |
|---|---|---|---|---|---|---|
| 9087975 | 6943962 | 5999332 | 10401940 | 25096940 | 1404551 | 8526183 |
| 11180780 | 9006505 | 7794482 | 19731400 | 46483320 | 2256264 | 30260230 |
| 8610404 | 6617012 | 3754382 | 8908109 | 19569870 | 1067030 | 13110040 |
| 9600308 | 8670389 | 5048034 | 10066280 | 25460920 | 1681620 | 9656001 |
| 7940868 | 8519054 | 6701782 | 11905000 | 16915160 | 1818939 | 9508179 |
| 8869575 | 9637784 | 21861090 | 25087400 | 25777990 | 3669539 | 31304850 |
| 9867292 | 8202416 | 5668906 | 12898990 | 29455690 | 2722045 | 19674080 |
| 10268480 | 8363442 | 4751304 | 11128520 | 29574220 | 1668853 | 11848780 |
| 7247256 | 5484054 | 2537280 | 4990730 | 12157840 | 369820.7 | 3188591 |
| 9758851 | 9152555 | 5961202 | 12900930 | 32325880 | 1994767 | 17905180 |
| 8553091 | 7476482 | 7242038 | 13951180 | 22102080 | 2275611 | 18249650 |
| 9352677 | 8012742 | 12598220 | 11423980 | 19338540 | 765306.8 | 6729850 |
| 8637384 | 7660878 | 7071890 | 18154120 | 25569660 | 3294949 | 26891610 |
| 7893192 | 5584488 | 3023387 | 5635348 | 17073220 | 534433.2 | 6897314 |
| 8352930 | 8176478 | 9418416 | 13471120 | 31464680 | 2558050 | 19825570 |
| 9881772 | 8122978 | 7309518 | 20280980 | 39164310 | 2601131 | 34644750 |
| 8387310 | 6910214 | 5381862 | 11723500 | 29521890 | 2182692 | 18963550 |
| 9085209 | 10571730 | 18600250 | 32854280 | 52514940 | 3886452 | 38436150 |
| 9980468 | 9610849 | 8260266 | 18184280 | 33209190 | 3566629 | 35826960 |
| 6911984 | 8761021 | 8020192 | 31358560 | 51173480 | 4954086 | 60545360 |
| 9661961 | 9012052 | 6006150 | 14170580 | 24180310 | 2432443 | 18016310 |
| 8585364 | 6561904 | 5042952 | 15148230 | 34029160 | 2286030 | 23742750 |
| 8237120 | 7016544 | 4019551 | 8404585 | 33699040 | 547262.2 | 5468246 |
| 9617844 | 8297940 | 10060320 | 20242720 | 32507330 | 5902326 | 39128690 |
| 7026116 | 6958582 | 4969080 | 16298710 | 29441300 | 3027614 | 34126110 |
| 4076615 | 3254037 | 2553640 | 4972984 | 10306660 | 605084.3 | 8419691 |
| 6531148 | 5642194 | 5041146 | 13027670 | 22120220 | 2950953 | 30231050 |
| 9755481 | 9610963 | 8745981 | 23362860 | 35729200 | 4698420 | 38168740 |
| 8684740 | 7801544 | 7471690 | 13808480 | 25808420 | 1939557 | 16207950 |
| 9822540 | 9606843 | 13534670 | 15929310 | 21233400 | 3339816 | 18798910 |

| Octadecenoi | Linoleate | Octadecatrie | Eicosatetrae | Eicosapentae | Docosahexae | (8Z-11Z-14Z) |
|---|---|---|---|---|---|---|
| 86026260 | 64366680 | 7323894 | 6038522 | 362588.4 | 3740096 | 735081.5 |
| 185299000 | 119672000 | 12219380 | 8445455 | 762599.2 | 2861897 | 1246639 |
| 106765800 | 66979870 | 6997794 | 4392152 | 364333.4 | 1088994 | 775873.2 |
| 87185740 | 45750580 | 5327236 | 3446544 | 392047.7 | 5144962 | 1170435 |
| 99471080 | 70724890 | 5140700 | 4027779 | 314620.5 | 2697774 | 1034542 |
| 170583400 | 109920800 | 12297790 | 7185906 | 1301600 | 6732830 | 1212279 |
| 106129100 | 84424760 | 8748765 | 6726430 | 899589.6 | 5356754 | 888305.2 |
| 99861250 | 49175270 | 5723974 | 3892483 | 356224.4 | 1855425 | 1463559 |
| 43294280 | 28346530 | 2511831 | 3676930 | 556564.2 | 1734119 | 562470.3 |
| 140798800 | 88723520 | 7952914 | 5372950 | 336893.6 | 1281345 | 948833.3 |
| 141462800 | 82836750 | 10212950 | 4754921 | 468774.7 | 3106027 | 1068908 |
| 70053430 | 43693270 | 4969792 | 3777862 | 695091.1 | 2898316 | 838683.1 |
| 167781300 | 99787460 | 9944348 | 4925868 | 514905.9 | 2774374 | 1479484 |
| 55310660 | 40318830 | 5212922 | 3554774 | 1309084 | 4902420 | 546228.6 |
| 131188700 | 111249700 | 9873033 | 8441828 | 470190.7 | 3747519 | 1939874 |
| 281566000 | 178340400 | 14776360 | 13411580 | 612894.2 | 3919904 | 2124083 |
| 163455200 | 93137340 | 5716550 | 7952390 | 465585.4 | 2347236 | 1172551 |
| 385824600 | 238457400 | 27353940 | 13116770 | 1364333 | 8484167 | 2053386 |
| 269526300 | 204792000 | 14172600 | 4001058 | 380763.2 | 3882898 | 1274245 |
| 384718600 | 285420100 | 29249260 | 13639110 | 776143.5 | 4812784 | 2559570 |
| 165044400 | 85070120 | 7502454 | 4402172 | 324212.2 | 2299110 | 1614104 |
| 248443800 | 171682400 | 22985430 | 9115615 | 589284.8 | 4386498 | 1586644 |
| 100112400 | 58903750 | 7283112 | 4493272 | 834164.2 | 4093998 | 863139 |
| 218493700 | 110552600 | 13025180 | 9591831 | 688838.1 | 4324194 | 2529945 |
| 238178400 | 135504100 | 11309820 | 6396798 | 421246.8 | 9146630 | 1565808 |
| 115698800 | 59345920 | 5858528 | 3555755 | 1027264 | 4038436 | 682385 |
| 144078800 | 123492500 | 10877020 | 8574037 | 395366.2 | 2074519 | 1292326 |
| 226613400 | 133203000 | 11739520 | 5850222 | 567108.8 | 3656052 | 1315140 |
| 119914800 | 62884510 | 7485494 | 4417158 | 453624.3 | 2646341 | 812005.9 |
| 126216500 | 87887440 | 7607770 | 5119246 | 1334700 | 7386134 | 885966.6 |

| (5Z-8Z-11Z-1 | (7Z-10Z-13Z- | Bilirubin |
|---|---|---|
| 362588.4 | 588663.6 | 983740 |
| 762599.2 | 1008496 | 2269457 |
| 364333.4 | 452378.2 | 936059.9 |
| 392047.7 | 468926.8 | 1430897 |
| 314620.5 | 620047.9 | 760257.8 |
| 1301600 | 1243933 | 2296647 |
| 899589.6 | 1120850 | 1024236 |
| 356224.4 | 668576.1 | 1174283 |
| 556564.2 | 325138.9 | 833361.5 |
| 336893.6 | 734933.6 | 766294.3 |
| 468774.7 | 875022.5 | 1474191 |
| 695091.1 | 498935.7 | 782062.3 |
| 514905.9 | 896519.7 | 2147631 |
| 1309084 | 771833.8 | 1063103 |
| 470190.7 | 1179720 | 902327.5 |
| 612894.2 | 1623269 | 5316778 |
| 465585.4 | 740475.1 | 727093.1 |
| 1364333 | 1967610 | 1619619 |
| 380763.2 | 892465.5 | 1509198 |
| 776143.5 | 2441195 | 4008191 |
| 324212.2 | 648523.5 | 709637.8 |
| 589284.8 | 1339895 | 293391.2 |
| 834164.2 | 722533.2 | 1062903 |
| 688838.1 | 1590772 | 2508128 |
| 421246.8 | 1097835 | 705633.7 |
| 1027264 | 907161.5 | 823266.9 |
| 395366.2 | 1022844 | 889843.2 |
| 567108.8 | 829285.4 | 1860027 |
| 453624.3 | 577943.8 | 703473.8 |
| 1334700 | 1040791 | 1436700 |
